# Grayscale ultrasound radiomics for characterizing subpleural pulmonary lesions: a multicenter prospective study

**DOI:** 10.1186/s13244-026-02244-1

**Published:** 2026-04-25

**Authors:** Jiawei Yi, Xinyu Zhao, Ke Bi, Kaiwen Wu, Runhe Xia, Yuning Luo, Yi Li, Mengjun Shen, Yang Cong, Yi Zhang, Yin Wang

**Affiliations:** 1 Department of Ultrasonography, Shanghai Pulmonary Hospital, School of Medicine, Tongji University, Shanghai, China; 2 Department of Nuclear Medicine, Shanghai Pulmonary Hospital, School of Medicine, Tongji University, Shanghai, China

**Keywords:** Lung cancer, Radiomics, Grayscale ultrasound, Subpleural pulmonary lesions

## Abstract

**Objectives:**

To develop and validate a radiomics model based on grayscale ultrasound (GSUS) images for characterizing subpleural pulmonary lesions (SPLs).

**Materials and methods:**

In this prospective, multicenter study, 738 patients with CT-confirmed SPLs were enrolled from three institutions and assigned to training (*n* = 407), internal validation (*n* = 146), and external validation (*n* = 185) cohorts. A total of 1320 radiomics features were extracted from both lesion and perilesional regions on GSUS images. Feature selection was performed through intra- and inter-class correlation coefficients (ICCs) analyses, Pearson correlation analyses, and least absolute shrinkage and selection operator (LASSO) regression. Clinical–radiomics fusion models were subsequently constructed by integrating selected radiomics features with key clinical variables using multivariate logistic regression. Model performance was evaluated comprehensively using the area under the receiver-operating characteristic curve (AUC), sensitivity, specificity, F1-score, and additional diagnostic metrics.

**Results:**

Five predictive models were constructed based on clinical, radiologic, and radiomics features. Among them, the integrated model combining lesion-based radiomics with clinical variables achieved the best diagnostic performance, with AUCs of 0.884 (95% CI: 0.828–0.940) in the internal validation cohort and 0.848 (95% CI: 0.791–0.904) in the external validation cohort. Calibration and decision curve analyses demonstrated good model calibration and favorable clinical utility. The diagnostic accuracy of the model was comparable to that of experienced lung ultrasound radiologists.

**Conclusions:**

The GSUS-based radiomics model effectively differentiates between benign and malignant SPLs, demonstrating strong diagnostic performance and promising clinical applicability.

**Critical relevance statement:**

The proposed ultrasound-based radiomics model provides a reproducible, noninvasive decision-support tool for characterizing subpleural pulmonary lesions, offering particular value in patients for whom invasive procedures are unsuitable or in settings where CT or biopsy is not readily available.

**Key Points:**

- Accurate characterization of subpleural pulmonary lesions remains challenging using conventional imaging techniques.
- The grayscale ultrasound radiomics model achieved accuracy comparable to expert radiologists.
- This model provides a noninvasive and accessible tool when CT or biopsy is limited.

**Graphical Abstract:**

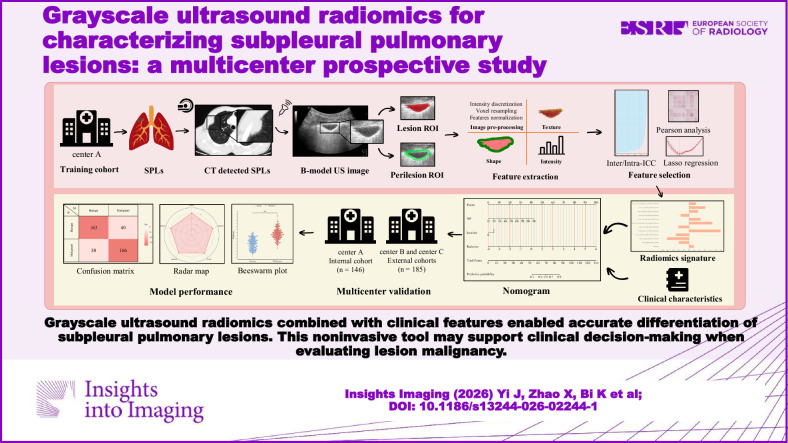

## Introduction

Subpleural pulmonary lesions (SPLs), which include over 90% of tuberculosis, 70%–80% of pneumonias, and approximately 40% of peripheral lung cancers, are often accessible via ultrasonography due to their proximity to the pleura and the absence of pulmonary air interference [1, 2]. Our previous studies [3–7] have demonstrated that conventional grayscale ultrasound (GSUS) and its advanced modalities, including contrast-enhanced ultrasound (CEUS) and elastography, hold promise for differentiating benign from malignant SPLs. However, the acquisition and interpretation of conventional ultrasound images remain highly operator-dependent and are influenced by subjective judgment and individual clinical experience. This variability hampers the reliable detection of subtle morphological and structural features, leading to challenges in reproducibility and inter-operator consistency. Even among experienced radiologists, such heterogeneity cannot be fully eliminated, thereby restricting the widespread clinical adoption and standardization of ultrasound-based assessment and diagnosis for SPLs.

Radiomics, a technique enabling high-throughput extraction of quantitative features from medical images, provides a noninvasive and reproducible approach for characterizing intralesional heterogeneity [8, 9]. This technique has been widely applied to CT in pulmonary research, where numerous CT-based radiomics models have demonstrated favorable performance in characterizing pulmonary nodules and distinguishing malignant from benign lesions [10–15]. In contrast, radiomics applications based on ultrasound remain relatively limited, particularly for SPL characterization, representing an emerging and underexplored domain.

Building on this foundation, the present study aims to evaluate the diagnostic potential of radiomics features extracted from GSUS images for the differentiation of benign and malignant SPLs. Furthermore, multiple predictive models integrating radiomics features with clinical variables were developed to evaluate their diagnostic robustness and clinical applicability. This approach seeks to offer a novel, noninvasive, and operator-independent strategy for the assessment of SPLs in clinical practice.

## Materials and methods

The study was approved by the Medical Ethical Committee of Shanghai Pulmonary Hospital (Approval No. K18-144), and written informed consent was obtained from all participants. The study was registered in the Chinese Clinical Trial Registry (ChiCTR1800019828).

### Study design

**Fig. 1:**
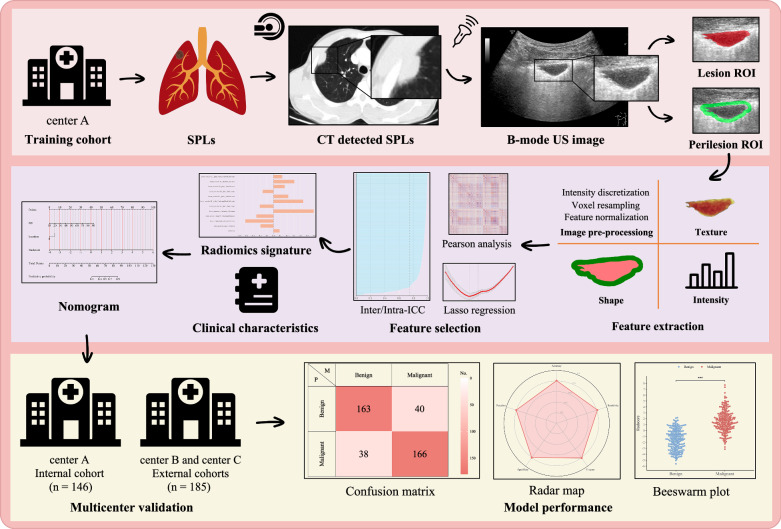
Workflow of the study design and radiomics analysis. SPLs, subpleural pulmonary lesions; CT, computed tomography; US, ultrasound; ROI, region of interest; Lasso, least absolute shrinkage and selection operator; ICC, inter-/intra-class correlation coefficients

**Fig. 2:**
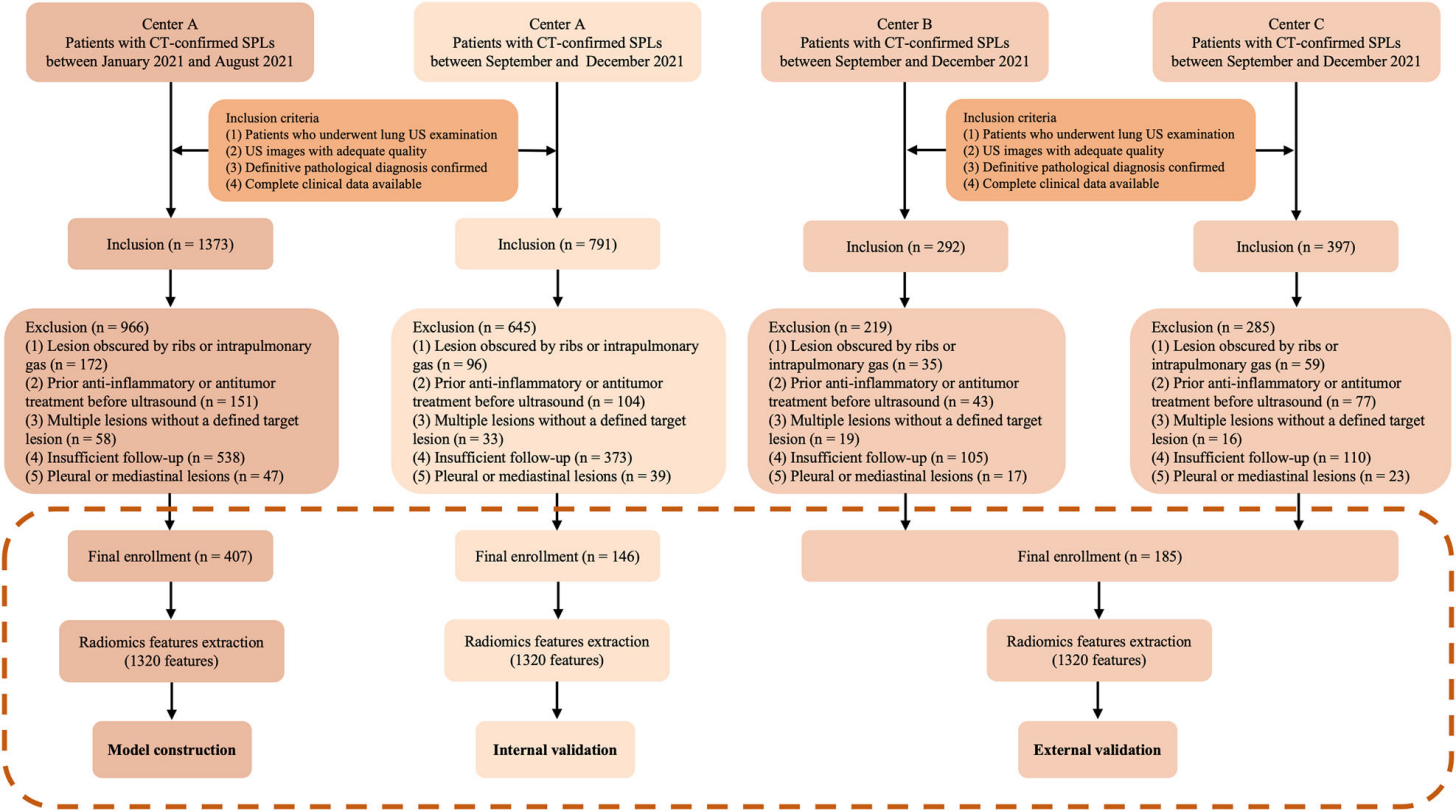
Workflow of the study. CT, computed tomography; SPLs, subpleural pulmonary lesions

The overall study design is illustrated in Fig. 1. This multicenter prospective study enrolled 738 patients with SPLs from three medical institutions. The training cohort (*n* = 407) and internal validation cohort (*n* = 146) were prospectively recruited from Center A between January and December 2021. The external validation cohort (*n* = 185) was prospectively recruited from Centers B and C between September and December 2021. The study workflow is presented in Fig. 2. Detailed inclusion and exclusion criteria are provided in Supplementary Material 1.

### US image acquisition and feature extraction

All lung ultrasound examinations were performed by radiologists formally trained in lung ultrasound. To ensure consistency across different centers, a standardized GSUS acquisition protocol was implemented. Although ultrasound equipment models differed, all examinations employed 1–6 MHz convex probes with unified imaging parameters (Table S1).

For each patient, dynamic ultrasound cine-loops were acquired. At least one high-quality GSUS image—clearly delineating the lesion and free from rib shadowing—was selected and stored in Digital Imaging and Communications in Medicine (DICOM) format to maintain consistency.

Image quality was independently evaluated by a senior radiologist with more than 15 years of experience in lung ultrasound. Subsequently, two radiologists (with 9 and 8 years of experience in lung ultrasound, respectively) manually delineated the lesion region of interest (ROI) using ITK-SNAP software (version 4.0) [16]. In addition to the primary lesion ROI, an annular perilesional ROI extending 5–10 mm beyond the lesion margin (adjusted based on lesion location and morphology) was manually defined to capture surrounding tissue characteristics [17]. Anatomical structures such as the chest wall, liver, heart, or spleen were meticulously excluded to maintain feature biological relevance and minimize confounding.

Prior to radiomics feature extraction, all GSUS images underwent speckle reduction preprocessing using the Speckle Reducing Anisotropic Diffusion (SRAD) algorithm [18–21]. Detailed preprocessing procedures are described in the Supplementary Material 2.

From the two ROIs (lesion and perilesional), 1320 radiomics features were extracted in total—660 from each region. Feature categories are summarized in Table S2. The entire radiomics pipeline complied with the Image Biomarker Standardization Initiative (IBSI) guidelines to ensure methodological transparency, reproducibility, and international standard alignment (Table S3) [22].

### Clinical and imaging data collection

Baseline demographic and clinical variables-including lesion size, location, pleural angle, margin clarity, pleural invasion, and comet tail sign-were collected. Final diagnoses were determined using a composite reference standard that incorporated histopathological results, microbiological testing, and follow-up imaging. Detailed definitions and diagnostic criteria are described in the Supplementary Material 3.

### Feature selection and Radscore construction

Radiomics features were standardized using z-score normalization to ensure comparability across features with different scales. To assess the reproducibility of manual segmentation, two radiologists independently performed manual segmentation on 50 randomly selected images.

Intra- and inter-observer agreement of the extracted radiomic features was evaluated using intra-and inter-observer class correlation coefficients (ICCs) [23]. Features demonstrating poor reliability (ICC < 0.75) were excluded from further analysis to ensure robustness of the selected features. Multicollinearity among features was addressed using Pearson correlation analysis [24]. Detailed procedures are provided in the Supplementary Material 4.

Feature selection was performed using least absolute shrinkage and selection operator (LASSO) logistic regression with a ten-fold cross-validation procedure to determine the optimal regularization parameter (λ). The λ_1se_ criterion was adopted to select a parsimonious feature set with minimal risk of overfitting. Selected features with non-zero coefficients were linearly combined to generate the radiomics score (Radscore), which served as an input for subsequent model development.

### Model development and validation

Based on the selected features, radiomics models were developed as follows: a lesion-only model was constructed using features extracted from lesion ROIs, while a lesion combined perilesional model was built by combining features from both lesion and perilesional ROIs. To further enhance diagnostic performance, clinical variables identified as independent predictors of malignancy through multivariate logistic regression were integrated with the Radscore to develop a clinical–radiomics fusion model. A nomogram was then constructed based on the fusion model for individualized prediction of the benign or malignant nature of SPLs.

All models were trained directly on the training cohort without parameter tuning, class weighting, oversampling, or undersampling. Model performance was subsequently evaluated independently on both the internal and external validation sets.

### Comparison with radiologists

To benchmark model performance against expert clinical interpretation, six board-certified radiologists with 5–15 years of lung ultrasound experience independently reviewed the independent test cohort (combined internal and external validation sets). Each radiologist assessed anonymized GSUS cine-loops to classify lesions as benign or malignant, blinded to clinical and pathological information. The specific evaluation prompt provided to radiologists is detailed in Supplementary Material 5.

To comprehensively assess model performance, model predictions were compared with each radiologist’s interpretation, as well as against the consensus diagnosis derived by majority vote among the six radiologists. This comparison aimed to evaluate the potential of the model as a clinical decision-support tool in distinguishing the nature of SPLs.

### Statistical analysis

Continuous variables were compared using the Kruskal–Wallis rank-sum test or the Mann–Whitney U test, as appropriate. Categorical variables were analyzed using the chi-square test. Logistic regression was employed to identify independent risk factors and construct the predictive models.

The discriminative performance of each model was evaluated using receiver-operating characteristic (ROC) curves and the corresponding area under the curve (AUC). Pairwise model comparisons were conducted using DeLong’s test. The optimal cutoff value for classification was identified using the Youden index. Subsequently, sensitivity, specificity, accuracy, precision, and the F1-score were calculated to assess classification performance comprehensively. McNemar’s test was applied to compare the diagnostic error rates between the model predictions and the radiologist consensus interpretation, to evaluate whether the differences were statistically significant.

All statistical tests were two-sided, with *p*-value < 0.05 considered statistically significant. Statistical analyses and model development were performed using R software (version 4.4) and Python (version 3.7).

## Results

### Baseline characters

The clinical and radiologic features of the three cohorts are summarized in Table 1. The distribution of benign versus malignant SPLs did not differ significantly among the training, internal validation, and external validation cohorts (*p* = 0.24), indicating a well-balanced and comparable case composition across datasets. Detailed comparisons between benign and malignant lesions within each cohort are presented in Table S4.

**Table 1:** Baseline clinical and imaging characteristics of patients

Characteristics	Training cohort (*n* = 407)	Internal validation cohort (*n* = 146)	External validation cohort (*n* = 185)	*p*-value
Sex (%)				0.68
Male	271 (66.6%)	98 (67.1%)	117 (63.2%)	
Female	136 (33.4%)	48 (32.9%)	68 (36.8%)	
Age (years, mean ± SD)	57.26 ± 16.06	60.67 ± 13.83	58.16 ± 13.00	**0.04**
Transverse diameter (mm, mean ± SD)	41.40 ± 24.23	40.77 ± 28.15	37.97 ± 22.66	0.19
Longitudinal diameter (mm, mean ± SD)	36.44 ± 18.57	34.12 ± 21.00	33.28 ± 19.78	**0.02**
Lesion size (%)				**0.02**
Small	134 (32.9%)	60 (41.1%)	81 (43.8%)	
Large	273 (67.1%)	86 (58.9%)	104 (56.2%)	
Location (%)				0.27
Upper/middle lobe	273 (67.1%)	89 (61%)	114 (61.6%)	
Lower lobe	134 (32.9%)	57 (39%)	71 (38.4%)	
Angle between lesion and pleura (%)				**0.01**
Acute	214 (52.6%)	98 (67.1%)	108 (58.4%)	
Obtuse	193 (47.4%)	48 (32.9%)	77 (41.6%)	
Margin (%)				**< 0.001**
Well-defined	212 (52.1%)	47 (32.2%)	88 (47.6%)	
Ill-defined	195 (47.9%)	99 (67.8%)	97 (52.4%)	
Pleural invasion (%)				0.09
Absent	239 (58.7%)	92 (63%)	126 (68.1%)	
Present	168 (41.3%)	54 (37%)	59 (31.9%)	
Comet tail sign (%)				**0.003**
Absent	170 (41.8%)	59 (40.4%)	103 (55.7%)	
Present	237 (58.2%)	87 (59.6%)	82 (44.3%)	
Radiologist (%)				0.66
Senior	191 (46.9%)	71 (48.6%)	81 (43.8%)	
Junior	216 (53.1%)	75 (51.4%)	104 (56.2%)	
Final diagnosis (%)				0.24
Benign	203 (49.9%)	61 (41.8%)	88 (47.6%)	
Malignant	204 (50.1%)	85 (58.2%)	97 (52.4%)	

Statistically significant *p*-values are bold

The Kruskal–Wallis rank-sum test was used to compare the difference in age, transverse diameter and longitudinal diameter on US. Chi-square test was used to compare the difference in sex, lesion size, location, angle between lesion and pleura, margin, pleural invasion and comet tail sign on US

### Model development

Model 1 was derived from a previously established prediction model incorporating five variables that have been independently validated as closely associated with malignant SPLs: age (× 1.000), obtuse angle between the lesion and pleura (× 19.231), well-defined lesion margin (× 14.646), presence of pleural invasion (× 48.385), and absence of comet tail sign (× 14.862). In the present study, this model was applied to all cohorts to assess its discriminative performance in differentiating between benign and malignant SPLs and served as a benchmark for comparative evaluation against other models (Fig. S1).

According to the predefined feature selection criteria, a total of 470 radiomic features and 940 combined features from the lesion and perilesional regions were retained for subsequent modeling (Supplementary Material 4; Figs. S2, S3).

Two radiomics models were constructed to predict the malignancy risk of SPLs: Model 2 was developed using features derived from the lesion region, while Model 3 incorporated both lesion and perilesional features. After LASSO regression for feature selection, 23 and 22 features were initially selected for Model 2 and Model 3, respectively. To optimize model simplicity and clinical applicability, iterative evaluation and comparison were conducted, resulting in the final selection of 11 features for Model 2 and 10 features for Model 3 (Tables S5, S6; Figs. S4, S5).

**Fig. 3:**
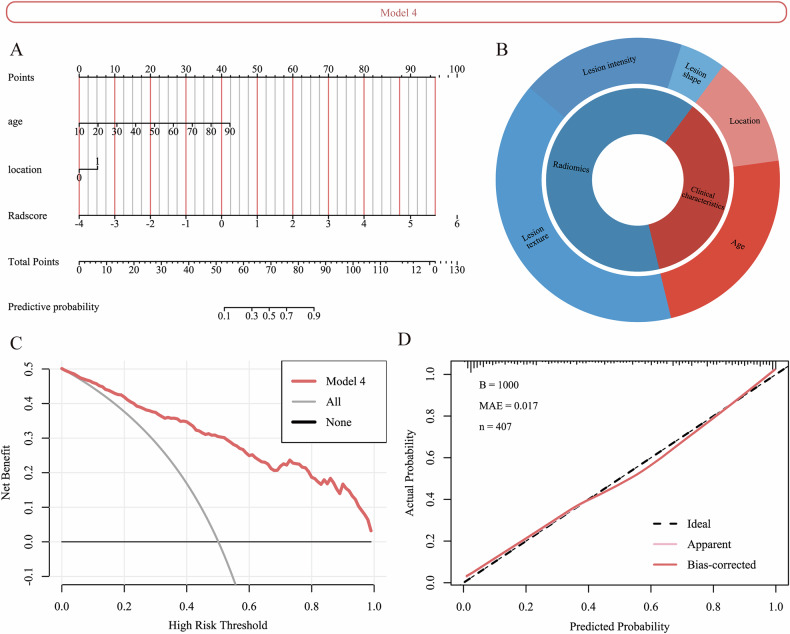
Construction and evaluation of Model 4. **A** The nomogram of Model 4, constructed based on Radscore and clinical features. **B** Relative contribution of different feature classes in the Model 4. **C** Decision curve analysis for Model 4, illustrating the net clinical benefit across a range of threshold probabilities. **D** Calibration curve assessing agreement between predicted and observed outcomes

Multivariate logistic regression analysis identified age and lesion location as independent clinical predictors of malignancy (Table 2). Based on these results, we combined the clinical variables with Radscores to develop two integrated models: Model 4, which incorporated the lesion-based Radscore and clinical variables, and Model 5, which incorporated a Radscore derived from both lesion and perilesional regions, together with the same clinical variables (Figs. 3, S6).

**Table 2:** Univariate and multivariate logistic regression analyses of Radscore and clinical variables

Variables	Univariate logistic regression analysis			Multivariate logistic regression analysis		
	OR	95% CI	*p-*value	OR	95% CI	*p-*value
Model 4						
Gender (Ref: female)	1.257	0.832–1.902	0.28			
Age^a^	1.090	1.070–1.113	**< 0.001**	1.074	1.051–1.099	**< 0.001**
Location (Ref: lower)^a^	1.892	1.245–2.891	**0.003**	2.003	1.122–3.618	**0.02**
Radscore^a^	4.682	3.464–6.549	**< 0.001**	3.829	2.800–5.434	**< 0.001**
Model 5						
Gender (Ref: female)	1.257	0.832–1.902	0.28			
Age^a^	1.090	1.070–1.113	**< 0.001**	1.076	1.053–1.102	**< 0.001**
Location (Ref: lower)^a^	1.892	1.245–2.891	**0.003**	1.971	1.102–3.566	**0.02**
Radscore^a^	5.756	4.117–8.350	**< 0.001**	4.755	3.332–7.065	**< 0.001**

Variables	Coef	OR	95% CI	*p*-value
Model 4				
Age	0.071	1.074	1.051–1.099	**< 0.001**
Location (Ref: lower)	0.694	2.003	1.122–3.618	**0.02**
Radscore	1.343	3.829	2.800–5.434	**< 0.001**
Intercept	−4.655			**< 0.001**
Model 5				
Age	0.073	1.076	1.053–1.102	**< 0.001**
Location (Ref: lower)	0.679	1.971	1.102–3.566	**0.02**
Radscore	1.559	4.755	3.332–7.065	**< 0.001**
Intercept	−4.795			**< 0.001**

Statistically significant *p*-values are bold

^a^ Variables with *p* < 0.2 on univariable analysis were included in multivariable analysis

### Model performance and comparative analysis

**Fig. 4:**
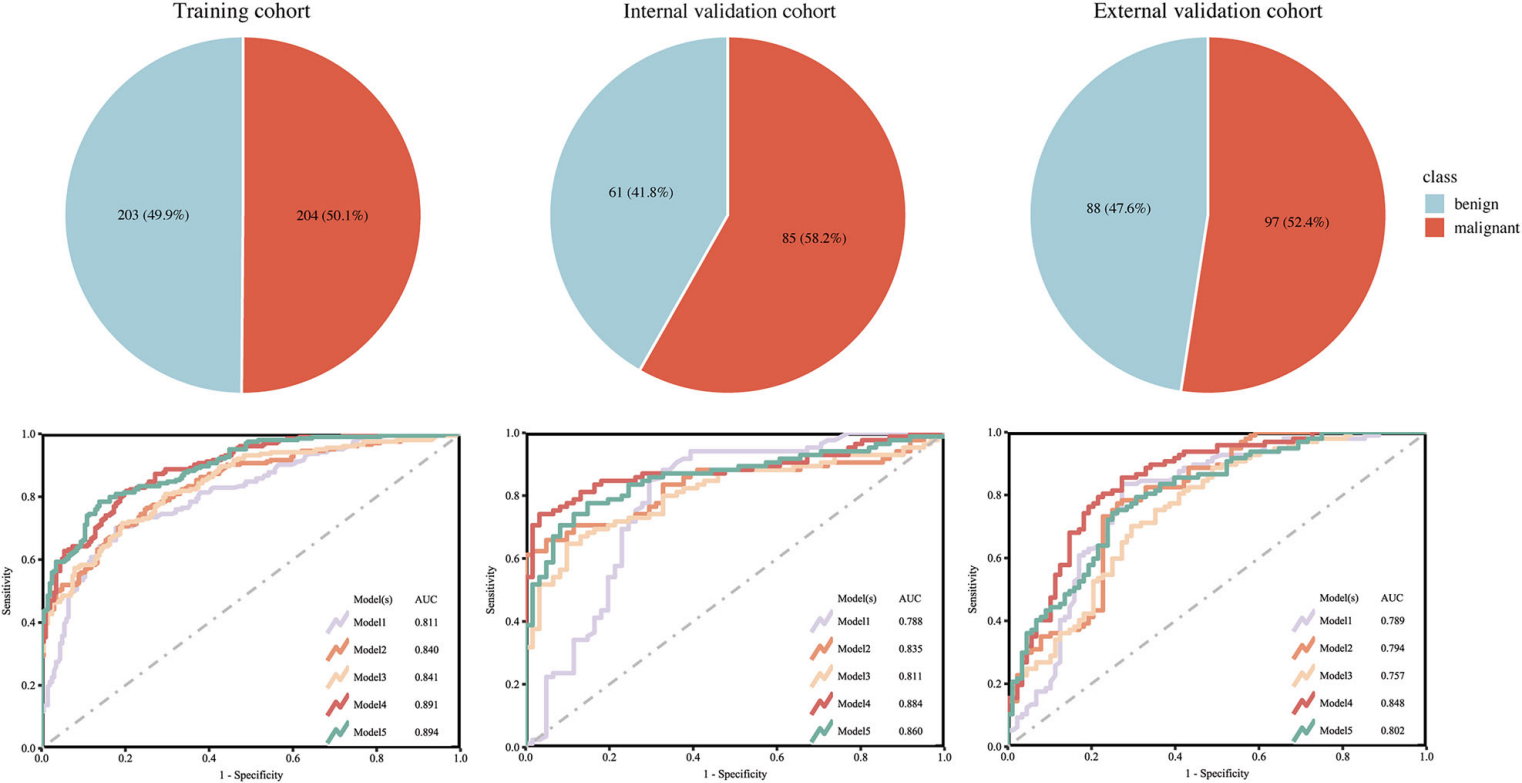
Case composition and diagnostic performance of five models across training, internal validation, and external validation cohorts. Pie charts display the distribution of benign and malignant cases in each cohort. AUC, area under the receiver-operating characteristic curve

**Fig. 5:**
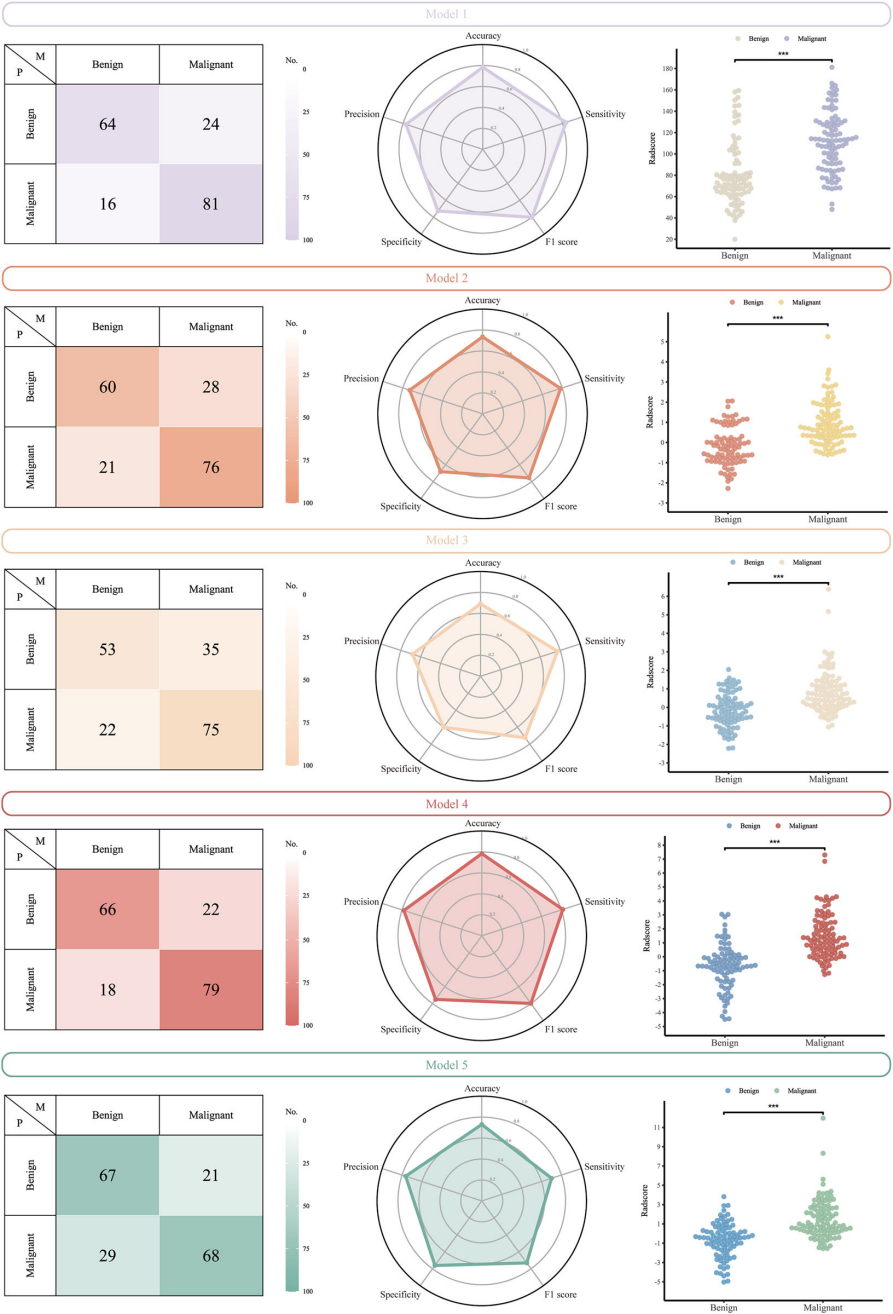
Comparative diagnostic performance of five models in the external validation cohort. Confusion matrices displaying classification results of the five models. Radar plots summarizing key diagnostic metrics (accuracy, sensitivity, specificity, precision, F1-score) of five models. Beeswarm plots showing the distribution of model-predicted scores between benign and malignant SPLs, with all models demonstrating statistically significant differences between the two groups. SPLs, subpleural pulmonary lesions

The discriminatory performance of the five models across three cohorts is summarized in Table 3 (Figs. 4, 5, S7, S8) Among them, Model 4 exhibited the most favorable diagnostic performance, achieving the highest AUC values in both validation cohorts: 0.884 (95% CI: 0.828–0.940) in the internal validation cohort and 0.848 (95% CI: 0.791–0.904) in the external validation cohort. DeLong’s test demonstrated the superior performance of Model 4 compared to the other models, with statistically significant improvements observed in most pairwise comparisons (*p* < 0.05) (Table S7). The calibration curves and decision curve analyses indicated good model calibration and favorable clinical utility (Figs. S9, S10).

**Table 3:** Comparative performance of five predictive models for differentiating benign and malignant SPLs

Model	Training cohort (*n* = 407)	Internal validation cohort (*n* = 146)	External validation cohort (*n* = 185)
Model 1 (Cutoff: 82.577)			
AUC	0.811 (0.770–0.852)	0.806 (0.731–0.882)	0.789 (0.721–0.858)
Sensitivity	0.765 (0.696–0.853)	0.800 (0.541–0.976)	0.835 (0.577–0.897)
Specificity	0.655 (0.576–0.818)	0.672 (0.541–0.820)	0.727 (0.500–0.807)
Accuracy	0.710	0.747	0.784
Precision	0.690	0.773	0.771
F1-score	0.726	0.786	0.802
Model 2 (Cutoff: 0.109)			
AUC	0.840 (0.802–0.877)	0.835 (0.768–0.901)	0.794 (0.729–0.859)
Sensitivity	0.701 (0.598–0.775)	0.765 (0.647–0.906)	0.784 (0.628–0.897)
Specificity	0.818 (0.719–0.877)	0.705 (0.557–0.951)	0.682 (0.534–0.830)
Accuracy	0.759	0.740	0.735
Precision	0.794	0.783	0.731
F1-score	0.745	0.774	0.756
Model 3 (Cutoff: 0.071)			
AUC	0.841 (0.803–0.879)	0.811 (0.741–0.881)	0.757 (0.688–0.826)
Sensitivity	0.716 (0.603–0.770)	0.812 (0.682–0.918)	0.773 (0.649–0.907)
Specificity	0.808 (0.685–0.867)	0.623 (0.426–0.803)	0.602 (0.489–0.750)
Accuracy	0.762	0.733	0.692
Precision	0.789	0.750	0.682
F1-score	0.751	0.780	0.725
Model 4 (Cutoff: 0.079)			
AUC	0.891 (0.862–0.921)	0.884 (0.828–0.940)	0.848 (0.791–0.904)
Sensitivity	0.814 (0.701–0.873)	0.859 (0.776–0.929)	0.814 (0.691–0.918)
Specificity	0.803 (0.700–0.862)	0.738 (0.311–0.934)	0.750 (0.625–0.864)
Accuracy	0.808	0.808	0.784
Precision	0.806	0.820	0.782
F1-score	0.810	0.839	0.798
Model 5 (Cutoff: 0.357)			
AUC	0.894 (0.864–0.923)	0.860 (0.799–0.920)	0.802 (0.739–0.864)
Sensitivity	0.784 (0.647–0.838)	0.800 (0.706–0.906)	0.701 (0.485–0.845)
Specificity	0.862 (0.719–0.916)	0.770 (0.590–0.934)	0.761 (0.648–0.841)
Accuracy	0.823	0.788	0.730
Precision	0.851	0.829	0.764
F1-score	0.816	0.814	0.731

Data in parentheses are 95% confidence intervals

*AUC* area under the receiver-operating-characteristics curve

To further explore the potential of simplifying the Radscore for practical application, the continuous Radscores from Models 4 and 5 were dichotomized using their respective optimal cutoff values, resulting in the development of binary-Radscore fusion models. In the validation cohorts, these simplified models maintained good diagnostic performance. Although the AUCs were slightly lower than those of the corresponding continuous-variable models, overall performance remained stable (Tables S8, S9; Figs. S11–S14).

### Comparison with radiologists

**Fig. 6:**
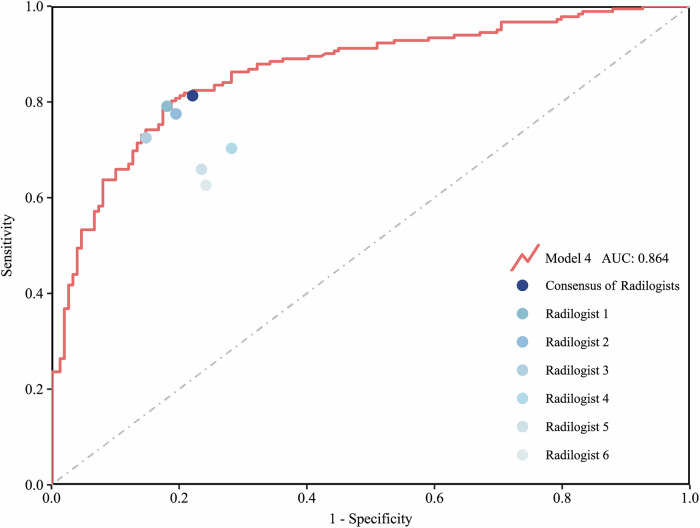
Performance comparison of Model 4 and radiologists. AUC, area under the receiver-operating characteristic curve

In the independent test cohort, Model 4 demonstrated robust discriminatory capability, achieving an AUC of 0.865, which was comparable to the subjective assessments made by radiologists. To further evaluate differences between Model 4 and expert clinical judgment, the consensus vote of the six radiologists was used as the reference standard (Fig. 6). McNemar’s test was performed to assess discrepancies in diagnostic error rates between Model 4 and the consensus interpretation (Table 4).

**Table 4:** Comparison of misclassification patterns between Model 4 and radiologist consensus

	Consensus of radiologists	
Model	**A:** number misclassified by neither 223	**B:** number misclassified by consensus but not by model 27
	**C:** number misclassified by model but not by consensus 41	**D:** number misclassified by both 40

The statistical analysis revealed no significant difference in diagnostic accuracy between Model 4 and the radiologist consensus (*p* > 0.05), suggesting that the model performs on par with the average expert-level clinical assessment (Tables S10; Figs. S15, S16).

### Subgroup analysis

We further conducted a subgroup analysis by pooling data from the training, internal validation, and external validation cohorts to evaluate the diagnostic performance of Model 4 across different clinical strata. The results demonstrated that the model maintained consistently robust performance in most subgroups, with AUC values exceeding 0.800. Notably, a slight decrease in discriminative ability was observed in the subgroup of patients with small lesions (maximum diameter < 3 cm), where the AUC was 0.787 (95% CI: 0.731–0.843) (Table S9; Fig. S17).

## Discussion

This study demonstrated the feasibility of GSUS-based radiomics for differentiating benign from malignant SPLs and developed a predictive model that integrates radiomics signatures with clinical variables. Given the widespread availability, low cost, and radiation-free nature of ultrasound, the proposed approach provides a practical and reproducible method for risk stratification, particularly for patients who are unsuitable for invasive procedures or require long-term surveillance. The GSUS-based radiomics approach proposed herein demonstrates substantial clinical utility in risk stratification of SPLs.

Five predictive models were constructed, and those integrating clinical variables (age and lesion location) consistently outperformed single-modality models across all validation cohorts. This highlights the complementary value of combining clinical information with radiomic signatures to better characterize lesion heterogeneity.

Notably, lesions located in the upper or middle lung lobes were associated with a higher malignancy risk, consistent with previous studies [25–27]. Several physiological mechanisms may explain this observation. First, perfusion in the upper lung regions is relatively low, and lymphatic drainage along the peribronchial system is slower, which may facilitate the deposition of inhaled particles, pathogens, or carcinogens, thereby increasing local tissue exposure and the likelihood of lesion development. Second, perfusion differences between upper and lower lung regions may influence the local immune microenvironment, angiogenesis, and tissue repair capacity, which could in turn affect tumor initiation, early growth, and invasive behavior. These physiological and microenvironmental factors may collectively contribute to the higher malignancy risk observed in upper or middle lung lobe lesions [27].

Among the models, Model 4 achieved the best overall performance in validation cohorts and maintained consistent accuracy across all subgroup analyses. To enhance practicality, the continuous Radscore was further dichotomized and re-modeled; the resulting binary-Radscore fusion model preserved strong discriminatory capability, highlighting its practical potential for routine clinical application.

To our knowledge, this is the first study to apply ultrasound-based radiomics to SPLs characterization, complementing existing CT-based radiomics research [10–15]. Our findings not only confirm the strong discriminative power of radiomics features from the lesion region but also assess the potential added value of perilesional features. However, in contrast to previous CT-based studies [17, 28–32], which reported the utility of perilesional regions, our findings revealed that incorporating perilesional features did not significantly improve model performance. This discrepancy may be attributable to the inherent limitations of ultrasound, where acoustic interference from aerated lung tissue and rib shadowing reduces perilesion visibility and compromises feature reliability [1]. Future studies focusing on cases with high-quality perilesion visualization may further clarify the potential value of these features.

We further compared the diagnostic performance of our model with that of experienced radiologists. In the independent test cohort, the model achieved accuracy comparable to that of senior readers. It is important to emphasize that the evaluation setting in this study was inherently more favorable to human readers than to the model. Specifically, radiologists interpreted dynamic cine-loops containing temporal information—such as lesion motion and respiratory artifacts—whereas the model relied solely on a static image and lacked access to this dynamic diagnostic information. In addition, radiologists were not constrained by time and were only required to perform binary classification without further subtype differentiation. These conditions collectively placed radiologists at a clear advantage. Despite this, the model still achieved performance comparable to expert interpretation, underscoring its potential clinical utility even when operating under substantially more limited informational input.

From a clinical translation perspective, this study exclusively utilized GSUS images for model development, deliberately excluding advanced modalities such as elastography and CEUS. This decision was guided by the goal of maximizing model accessibility and generalizability. As the most commonly used and widely available ultrasound modality, GSUS offers broad applicability, particularly in primary care settings and resource-limited environments. However, we acknowledge that reliance on a single imaging modality may constrain diagnostic ceiling. Future efforts will explore the integration of multimodal ultrasound techniques and the integration of CT-derived radiomic features to further enhance model accuracy and expand its clinical utility [33, 34].

Several limitations should be acknowledged. First, lesion ROI segmentation was manually performed. Although considered the gold standard, manual delineation is time-consuming and subject to operator variability. Incorporating automated segmentation methods (e.g., nnU-Net) in future work may improve reproducibility and efficiency [35]. Second, the diagnostic performance of the model declined for lesions smaller than 3 cm. This limitation is likely inherent to the evaluation of very small SPLs, in which restricted texture information, increased boundary ambiguity, and the spatial resolution constraints of ultrasound reduce the amount of reliable quantitative information available for radiomic analysis. Future studies with larger datasets enriched for small lesions may enable the development of models specifically optimized for this subgroup. Moreover, the use of higher-resolution ultrasound and the incorporation of multimodal imaging—such as CT—could further enhance the model’s ability to characterize micro-lesions. Finally, the model’s interpretability remains limited. The high-dimensional radiomic features function as “black-box” variables and lack clear associations with underlying biological or molecular mechanisms. This may hinder clinical acceptance and limit translational potential. To address this, future studies will aim to incorporate multi-omics data, including genomics, transcriptomics, and proteomics, to better elucidate the molecular basis of radiomic phenotypes and enhance the biological interpretability and clinical relevance of the predictive model [36–38].

In summary, this study provides preliminary evidence that radiomics features extracted from GSUS images are closely associated with the malignancy status of SPLs. The proposed model offers a noninvasive and cost-effective tool to assist clinical decision-making, with the potential to serve as a surrogate diagnostic approach in cases where histopathological sampling is not feasible. This GSUS-based radiomics strategy represents a valuable complement to existing diagnostic pathways and holds promise for enhancing the precision management of SPLs.

## Supplementary information

ELECTRONIC SUPPLEMENTARY MATERIAL

## Data Availability

The datasets used and/or analyzed during the current study are available from the corresponding author on reasonable request.

## Abbreviations

AUC: Area under the receiver-operating characteristic curve
CEUS: Contrast-enhanced ultrasound
GSUS: Grayscale ultrasound
ICCs: Intra-/inter-class correlation coefficients
LASSO: Least absolute shrinkage and selection operator
Radscore: Radiomics score
ROI: Region of interest
SPLs: Subpleural pulmonary lesions
