# Supplementary material for: Grayscale ultrasound radiomics for characterizing subpleural pulmonary lesions: a multicenter prospective study
Source: Insights Imaging. 2026 Apr 25;17:117. doi: 10.1186/s13244-026-02244-1 (PMC13110262; doi:10.1186/s13244-026-02244-1)

Grayscale Ultrasound Radiomics for Characterizing Subpleural Pulmonary Lesions: A Multicenter Prospective Study

ELECTRONIC SUPPLEMENTARY MATERIAL

Table of Contents

Supplementary Material 1 Inclusion and Exclusion Criteria for the Three Study Cohorts (Page 4)
Supplementary Material 2 Adaptive Speckle Noise Reduction Using Anisotropic Diffusion for Image Preprocessing (Page 5)
Supplementary Material 3 Clinical and Imaging Data Collection and Diagnostic Criteria (Page 7)
Supplementary Material 4 Workflow and Results of Radiomic Feature Selection (Page 9)
Supplementary Material 5 Radiologist Comparison Prompt (Page 11)
Table S1 Standardized Ultrasound Imaging Protocol
Table S2 Radiomics Features Extracted from Lesion ROI and Perilesion ROI
Table S3 Imaging Biomarker Standardization Initiative (IBSI) Reporting Structure of the Study
Table S4 Comparison of Clinical and Ultrasonographic Features between Benign and Malignant SPLs across Cohorts
Table S5 Feature Coefficients Used for Constructing the Model 2 Radscore
Table S6 Feature Coefficients Used for Constructing the Model 3 Radscore
Table S7 Statistical Evaluation of Model Discrimination
Table S8 Univariable and Multivariable Logistic Regression Analyses of Categorized Radscore and Clinical Variables
Table S9 Predictive Performance of Models Using Categorical Radscore Derived from Model 4 and Model 5
Table S10 Diagnostic Performance Metrics of Individual Radiologists and Consensus Interpretation
Table S11 Subgroup Analysis of Model Performance
Figure S1 Construction and evaluation of Model 1
Figure S2 Inter- and intra-observer reproducibility of radiomic feature extraction
Figure S3 Pearson correlation analysis of radiomic features
Figure S4 Construction and evaluation of Model 2
Figure S5 Construction and evaluation of Model 3
Figure S6 Construction and evaluation of Model 5
Figure S7 Comparative diagnostic performance of five models in the training cohort
Figure S8 Comparative diagnostic performance of five models in the internal validation cohort
Figure S9 Decision and calibration curve analysis of five models in the internal validation cohort
Figure S10 Decision and calibration curve analysis of five models in the external validation cohort
Figure S11 Construction and evaluation of the binary-Radscore Model 4
Figure S12 Construction and evaluation of the Binary-Radscore Model 5
Figure S13 Diagnostic performance comparison of two Binary-Radscore models (Model 4 and Model 5) in the internal validation cohort

Figure S14 Diagnostic performance comparison of two Binary-Radscore models (Model 4 and Model 5) in the external validation cohort

Figure S15 Inter-reader agreement among six radiologists on the independent test cohort

Figure S16 Diagnostic performance of six radiologists on the independent test cohort

Figure S17 Diagnostic performance of Model 4 across different subgroups

Inclusion and exclusion criteria for the three study cohorts

Patients were prospectively enrolled between January and December 2021 across three medical institutions. Patients were included if they met all of the following criteria: (1) presence of subpleural pulmonary lesions (SPLs) confirmed by chest CT and subsequently evaluated by lung ultrasound; (2) availability of high-quality grayscale ultrasound (GSUS) images without significant motion or artifact interference; (3) a definitive diagnosis established via surgical resection, needle biopsy, microbiological testing, or long-term imaging and clinical follow-up; and (4) complete clinical data without significant omissions. Exclusion criteria were as follows: (1) poor lesion visualization on ultrasound due to rib shadowing or intrapulmonary gas; (2) prior use of anti-inflammatory or antitumor therapy before the ultrasound examination; (3) presence of multiple lesions without a clearly defined target lesion; (4) follow-up duration of less than two years or lack of a definitive outcome; and (5) lesions located outside the pulmonary parenchyma, such as those in the pleura or mediastinum. All criteria were consistently applied across participating centers to ensure standardization of the study population.

Adaptive speckle noise reduction using anisotropic diffusion for image preprocessing

To minimize the interference of speckle noise on radiomics feature extraction from GSUS images, all images underwent preprocessing using an adaptive Speckle Reducing Anisotropic Diffusion (SRAD) algorithm prior to feature extraction. This nonlinear, edge-preserving diffusion technique—optimized for GSUS image enhancement—adjusts the diffusion intensity adaptively based on local image gradients. This approach effectively smooths the image while preserving anatomical edge details.

In this study, the global noise level of each image was first estimated, and the mean noise intensity (σ) was used to guide the dynamic adjustment of key diffusion parameters, including the number of iterations (n_iter), gradient threshold (kappa), and diffusion coefficient (gamma). This adaptive strategy balanced noise suppression and structural fidelity under varying noise conditions. Specifically, for low-noise images ($\sigma \leq 0.4$), where edge details remain clear, a lower diffusion strength was applied to preserve fine structures (n_iter = 15, kappa = 20, gamma = 0.15). For moderate-noise images ($0.4 < \sigma \leq 0.7$), where noise is more prominent but edge information remains discernible, enhanced

diffusion was employed to improve denoising effectiveness (n_iter = 20, kappa = 25, gamma = 0.20). For high-noise images ($\sigma > 0.7$), characterized by substantial speckle artifacts, stronger diffusion was applied to suppress noise while adjusting kappa and gamma to maintain edge integrity (n_iter = 25, kappa = 35, gamma = 0.25). This adaptive SRAD protocol significantly improved image quality and consistency, facilitating more robust radiomic feature extraction and enhancing model predictive performance and reproducibility.

Clinical and imaging data collection and diagnostic criteria

Comprehensive clinical data were systematically collected for all enrolled patients, including sex, age, anatomical location of the lesion (classified as upper/middle lobe vs. lower lobe), and operator experience level, which was stratified based on years of lung ultrasound practice into junior (<5 years) and senior (≥5 years) groups. The radiologic features of each lesion in GSUS were meticulously evaluated and included the following parameters: (1) Transverse diameter: the maximum diameter of the lesion parallel to the chest wall; (2) Longitudinal diameter: the maximum diameter perpendicular to the chest wall; (3) Lesion size: classified as small (<3 cm) or large (≥3 cm) based on the maximum transverse or longitudinal diameter; (4) The angle between the lesion and pleura: lesions were considered to form an obtuse angle if any angle between the lesion and the pleura exceeded 90°; (5) Lesion margin clarity: assessed as either well-defined or ill-defined; (6) Pleural invasion: defined as the presence of localized pleural thickening adjacent to the lesion; (7) Comet-tail sign: defined as the presence of typical hyperechoic reverberation artifacts posterior to the lesion.

The final diagnosis was based on a comprehensive evaluation integrating histopathological, microbiological, and imaging follow-

up findings. All SPLs were diagnosed through tissue sampling obtained via core needle biopsy or surgical resection. Specimens were fixed in 10% neutral formalin, routinely processed for hematoxylin and eosin (H&E) staining, and subjected to immunohistochemical analysis to confirm the pathological nature of the lesions. For cases lacking definitive pathological diagnosis, two senior pulmonologists reached consensus by reviewing the imaging data, microbiological test results, and clinical presentation to establish a diagnostic classification. All patients were followed for a minimum of 2 years to ensure diagnostic stability and confirmatory accuracy.

Radiomic feature selection workflow and results

In this study, all initially extracted radiomic features were systematically screened to ensure that the features included in model construction were both stable and independent.

To assess feature stability, we calculated intra-class correlation coefficients (intra-ICC) for intra-observer and inter-class correlation coefficients (inter-ICC). Two radiologists (M.J.S. and Y.Z., with 9 and 8 years of lung ultrasound reading experience, respectively) randomly selected 50 ultrasound images and manually delineated regions of interest (ROIs) for both the lesion and perilesion areas. Radiomic features were then extracted from these ROIs. Inter-ICC was used to evaluate reproducibility across observers. For intra-observer consistency, one radiologist (M.J.S.) repeated the ROI delineation and feature extraction on the same 50 images after two months. Intra-observer ICC was then calculated to assess repeatability. Features with both intra- and inter-ICC ≥ 0.75 were considered reproducible and retained for subsequent analysis.

The results showed that, for lesion features, the mean intra-ICC was 0.94 (range, 0.05–1.00) and the mean inter-ICC was 0.91 (range, 0.02–1.00). For perilesion features, the mean intra-ICC was 0.98 (range, 0.08–1.00) and the mean inter-ICC was 0.86 (range, 0.01–0.99). Based on these criteria, 39 lesion features

(5.9% of all lesion features) and 92 perilesion features (13.9% of all perilesion features) were excluded (Fig. S2).

To further reduce redundancy and potential multicollinearity, Pearson correlation analysis was performed among the remaining features. For highly correlated feature pairs ($r > 0.9$), only the feature with greater representativeness or clinical relevance was retained, and the other was removed (Fig. S3).

Following this screening process, 470 lesion-derived features and 940 combined features from lesion and perilesion regions were retained. All included features demonstrated high stability and low inter-feature correlation, making them suitable for subsequent model construction.

Radiologist comparison prompt

Each radiologist was provided with the following standardized instruction, along with anonymized dynamic GSUS cine-loops for independent evaluation.

“This multicenter, prospective study developed an ultrasound-based radiomics model to differentiate benign from malignant subpleural pulmonary lesions (SPLs) in patients with CT-confirmed SPLs who subsequently underwent grayscale ultrasound (GSUS). We cordially invite you to participate in the reader study designed to benchmark radiologist performance against the model.

The assessment set comprises approximately 300 GSUS cine-loops of SPLs collected from three tertiary centers. All lesions are clearly visualized, and the image quality meets predefined standards.

For each case, please review the full dynamic GSUS video and determine whether the lesion exhibits imaging features suggestive of malignancy. You are encouraged to interpret the images exactly as you would in routine clinical practice. No ancillary clinical or imaging information will be provided. We acknowledge the inherent limitations of a single-modality assessment and therefore rely on your professional expertise and judgement.

After all readings have been completed, diagnostic performance will be compared with that of the radiomics model, with a primary focus on the accuracy of malignancy classification in SPLs.

We appreciate your participation and look forward to sharing the study results with you!”

Table S1 Standardized Ultrasound Imaging Protocol

**Ultrasound image acquisition**	
Scanner model	LOGIQ E9 / LQGIQ E20 (General Electric Healthcare, Chicago, IL, USA)
Probe type	Convex probe
Frequency	1-6 MHz (adjusted based on patient habitus)
Imaging depth	0-10 cm
Gain	Adjusted to optimize pleural line visualization; no automatic gain control was used
Time gain compensation (TGC)	Set to maintain uniform brightness from pleura to subpleural region
Focus position	below the pleural line
Harmonic imaging	off (to preserve artifact fidelity)
Image export format	DICOM
Examiner	All scans performed by radiologists with ≥ 3 years of experience in lung ultrasound
Probe contact	Probe placed perpendicular to the rib space to optimize pleural line visualization, avoiding rib shadowing
**Patient preparation and positioning**	
Position	Patients were positioned in a sitting, supine, lateral, or prone posture depending on the location of the lesion.
Breath control	Patients were instructed to hold their breath briefly during image acquisition when possible
Skin preparation	Ultrasound gel applied directly; no additional preparation was required
Fasting required	Not required

Table S2 Radiomics Features Extracted from Lesion ROI and Perilesion ROI

Feature classes	Feature names	ROIs
Original features		
Shape-based (2D)	Elongation, MajorAxisLength, MaximumDiameter, MeshSurface, MinorAxisLength, Perimeter, PerimeterSurfaceRatio	Lesion, Perilesion
First-order Statistics	10Percentile, 90Percentile, Energy, Entropy, InterquartileRange, Kurtosis, Maximum, MeanAbsoluteDeviation, Mean, Median, Minimum, Range, RobustMeanAbsoluteDeviation, RootMeanSquared, Skewness, TotalEnergy, Uniformity, Variance	Lesion, Perilesion
Grey Level Co-occurrence Matrix (GLCM)	Autocorrelation, ClusterProminence, ClusterShade. ClusterTendency, Contrast, Correlation, DifferenceAverage, DifferenceEntropy, DifferenceVariance, Id, Idm, Idmn, Idn, Imc1, Imc2, InverseVariance, JointAverage, JointEnergy, JointEntropy, MCC, MaximumProbability, SumAverage, SumEntropy, SumSquares	Lesion, Perilesion
Grey Level Run Length Matrix (GLRLM)	GrayLevelNonUniformity, GrayLevelNonUniformityNormalized, GrayLevelVariance, HighGrayLevelRunEmphasis, LongRunEmphasis, LongRunHighGrayLevelEmphasis, LongRunLowGrayLevelEmphasis, LowGrayLevelRunEmphasis, RunEntropy, RunLengthNonUniformity, RunLengthNonUniformityNormalized, RunPercentage, RunVariance, ShortRunEmphasis, ShortRunHighGrayLevelEmphasis, ShortRunLowGrayLevelEmphasis	Lesion, Perilesion
Grey Level Size Zone Matrix (GLSZM)	GrayLevelNonUniformity, GrayLevelNonUniformityNormalized, GrayLevelVariance, HighGrayLevelZoneEmphasis, LargeAreaEmphasis, LargeAreaHighGrayLevelEmphasis, LargeAreaLowGrayLevelEmphasis, LowGrayLevelZoneEmphasis, SizeZoneNonUniformity, SizeZoneNonUniformityNormalized, SmallAreaEmphasis, SmallAreaHighGrayLevelEmphasis, SmallAreaLowGrayLevelEmphasis, ZoneEntropy, ZonePercentage, ZoneVariance	Lesion, Perilesion

Grey Level Dependence Matrix (GLDM)	DependenceEntropy, DependenceNonUniformity, DependenceNonUniformityNormalized, DependenceVariance, GrayLevelNonUniformity, GrayLevelVariance, HighGrayLevelEmphasis, LargeDependenceEmphasis, LargeDependenceHighGrayLevelEmphasis, LargeDependenceLowGrayLevelEmphasis, LowGrayLevelEmphasis, SmallDependenceEmphasis, SmallDependenceHighGrayLevelEmphasis, SmallDependenceLowGrayLevelEmphasis	Lesion, Perilesion
Neighbouring Grey Tone Difference Matrix (NGTDM)	Busyness, Coarseness, Complexity, Contrast, Strength	Lesion, Perilesion
Wavelet features	wavelet(LL)_x, wavelet(LH)_x, wavelet(HL)_x, wavelet(HH)_x	Lesion, Perilesion
Gradient features	gradient_x	Lesion, Perilesion
Squareroot features	squareroot_x	Lesion, Perilesion

ROI = region of interest.

Table S3 Imaging Biomarker Standardization Initiative (IBSI) Reporting Structure of the Study

Parameter category	Value / Setting
**Delineation**	
Software	ITK-SNAP software (version 4.0; www.itksnap.org)
ROI definition	Standard 2D ROI tools
ROI delineation method	Manual delineation by two radiologists
ROI format	Binary mask
Imaging type	Ultrasound (grayscale)
**Radiomics feature extraction**	
Software	Python (version 3.11)
Package	PyRadiomics v3.0
Discretization	Fixed bin width
Resampling method	Linear interpolation
Resampled voxel size	1.0 × 1.0 mm (2D)
Bin width	25
Types of features extracted	First-order, Shape, Grey Level Co-occurrence Matrix (GLCM), Grey Level Run Length Matrix (GLRLM), Grey Level Size Zone Matrix (GLSZM), Grey Level Dependence Matrix (GLDM),Neighbouring Grey Tone Difference Matrix (NGTDM), wavelet (LL)_x, wavelet (LH)_x, wavelet (HL)_x, wavelet (HH)_x, gradient_x, squareroot_x
Normalization	Z-score
Inter-/intra-observer reproducibility	ICC analysis
Exclusion criteria	ICC smaller than 0.75

ROI = region of interest, ICC = Inter-/intra-class Correlation Coefficient.

Table S4 Comparison of Clinical and Ultrasonographic Features between Benign and Malignant SPLs across Cohorts

**Characteristic**	**Training cohort (n = 407)**			**Internal test cohort (n = 146)**			**External test cohort (n = 185)**		
	**Benign (n = 203)**	**Malignant (n = 204)**	***p***	**Benign (n = 61)**	**Malignant (n = 85)**	***p***	**Benign (n = 88)**	**Malignant (n = 97)**	***p***
Sex (%)			0.33			0.38			0.21
Male	130 (64.0%)	141 (69.1%)		38 (62.3%)	60 (70.6%)		51 (58.0%)	66 (68.0%)	
Female	73 (36.0%)	63 (30.9%)		23 (37.7%)	25 (29.4%)		37 (42.0%)	31 (32.0%)	
Age (year, mean ± SD)	49.16 ± 17.12	65.31 ± 9.67	**< 0.001**	53.74 ± 15.28	65.65 ± 10.18	**< 0.001**	52.68 ± 13.71	63.13 ± 10.06	**< 0.001**
Transverse diameter (mm, mean ± SD)	32.94 ± 18.31	49.83 ± 26.41	**< 0.001**	28.66 ± 16.64	49.47 ± 31.41	**< 0.001**	30.09 ± 17.36	45.11 ± 24.55	**< 0.001**
Longitudinal diameter (mm, mean ± SD)	31.75 ± 16.14	41.12 ± 19.65	**< 0.001**	24.18 ± 13.43	41.25 ± 22.56	**< 0.001**	26.02 ± 14.43	39.87 ± 21.66	**< 0.001**
Lesion size (%)			**< 0.001**			**< 0.001**			**0.003**
Small	86 (42.4%)	48 (23.5%)		39 (63.9%)	21 (24.7%)		49 (55.7%)	32 (33.0%)	
Large	117 (57.6%)	156 (76.5%)		22 (36.1%)	64 (75.3%)		39 (44.3%)	65 (67.0%)	
Location (%)			**0.004**			0.91			**0.03**
Upper/middle lobe	122 (60.1%)	151 (74.0%)		38 (62.3%)	51 (60%)		62 (70.5%)	52 (53.6%)	
Lower lobe	81 (39.9%)	53 (26.0%)		23 (37.7%)	34 (40%)		26 (29.5%)	45 (46.4%)	
Angle between lesion and pleura (%)			**< 0.001**			0.10			**< 0.001**
Acute	130 (64.0%)	84 (41.2%)		46 (75.4%)	52 (61.2%)		63 (71.6%)	45 (46.4%)	
Obtuse	73 (36.0%)	120 (58.8%)		15 (24.6%)	33 (38.8%)		25 (28.4%)	52 (53.6%)	
Margin (%)			**0.02**			**0.03**			0.11
Well-defined	93 (45.8%)	119 (58.3%)		13 (21.3%)	34 (40%)		36 (40.9%)	52 (53.6%)	

ill-defined	110 (54.2%)	85 (41.7%)		48 (78.7%)	51 (60%)		52 (59.1%)	45 (46.4%)	
Pleural invasion (%)			**< 0.001**			**0.002**			**< 0.001**
Absent	157 (77.3%)	82 (40.2%)		48 (78.7%)	44 (51.8%)		72 (81.8%)	54 (55.7%)	
Present	46 (22.7%)	122 (59.8%)		13 (21.3%)	41 (48.2%)		16 (18.2%)	43 (44.3%)	
Comet tail sign (%)			0.73			**0.04**			0.46
Absent	87 (42.9%)	83 (40.7%)		18 (29.5%)	41 (48.2%)		46 (52.3%)	57 (58.8%)	
Present	116 (57.1%)	121 (59.3%)		43 (70.5%)	44 (51.8%)		42 (47.7%)	40 (41.2%)	
Radiologist (%)			0.96			0.70			0.76
Senior	96 (47.3%)	95 (46.6%)		28 (45.9%)	43 (50.6%)		37 (42%)	44 (45.4%)	
Junior	107 (52.7%)	109 (53.4%)		33 (54.1%)	42 (49.4%)		51 (58%)	53 (54.6%)	

SD = standard deviation.

Statistically significant p values are bold.

Table S5 Feature Coefficients used for Constructing the Model 2 Radscore

Features	Standardized Coefficients
Intercept	0.0897
lesion_squareroot_firstorder_Maximum	0.5942
lesion_wavelet-HL_glrlm_RunLengthNonUniformity	0.4398
lesion_original_glcm_InverseVariance	-0.4168
lesion_wavelet-LL_firstorder_Kurtosis	0.3071
lesion_original_shape2D_MaximumDiameter	-0.2528
lesion_wavelet-HL_glszm_ZoneVariance	0.2177
lesion_wavelet-HL_glrlm_RunEntropy	-0.2024
lesion_gradient_ngtdm_Contrast	-0.1923
lesion_wavelet-LH_glszm_ZoneVariance	0.1655
lesion_wavelet-LH_glrlm_RunEntropy	-0.16
lesion_wavelet-LL_gldm_GrayLevelNonUniformity	0.1292

Radscore = 0.0897 + lesion_squareroot_firstorder_Maximum*0.5942 + lesion_wavelet-HL_glrlm_RunLengthNonUniformity*0.4398 + lesion_original_glcm_InverseVariance*-0.4168 + lesion_wavelet-LL_firstorder_Kurtosis*0.3071 + lesion_original_shape2D_MaximumDiameter*-0.2528 + lesion_wavelet-HL_glszm_ZoneVariance*0.2177 + lesion_wavelet-HL_glrlm_RunEntropy*-0.2024 + lesion_gradient_ngtdm_Contrast*-0.1923 + lesion_wavelet-LH_glszm_ZoneVariance*0.1655 + lesion_wavelet-LH_glrlm_RunEntropy*-0.16 + lesion_wavelet-LL_gldm_GrayLevelNonUniformity*0.1292

Table S6 Feature Coefficients Used for Constructing the Model 3 Radscore

**Features**	**Standardized Coefficients**
(Intercept)	0.0348
lesion_wavelet-LL_glcm_Idn	0.3283
lesion_wavelet-LL_gldm_GrayLevelNonUniformity	0.2927
lesion_original_glcm_InverseVariance	-0.2815
peri_wavelet-HL_glcm_Imc2	-0.2731
peri_wavelet-LL_firstorder_90Percentile	0.2262
peri_original_firstorder_Maximum	0.2012
lesion_wavelet-HL_glrlm_RunLengthNonUniformity	0.1639
peri_wavelet-LL_gldm_SmallDependenceLowGrayLevelEmphasis	0.1582
peri_squareroot_gldm_GrayLevelNonUniformity	-0.1454
lesion_wavelet-HL_glszm_ZoneVariance	0.1385

Radscore = 0.0348 + lesion_wavelet-LL_glcm_Idn*0.3283 + lesion_wavelet-LL_gldm_GrayLevelNonUniformity*0.2927 + lesion_original_glcm_InverseVariance*-0.2815 + peri_wavelet-HL_glcm_Imc2*-0.2731 + peri_wavelet-LL_firstorder_90Percentile*0.2262 + peri_original_firstorder_Maximum*0.2012 + lesion_wavelet-HL_glrlm_RunLengthNonUniformity*0.1639 + peri_wavelet-LL_gldm_SmallDependenceLowGrayLevelEmphasis*0.1582 + peri_squareroot_gldm_GrayLevelNonUniformity*-0.1454 + lesion_wavelet-HL_glszm_ZoneVariance*0.1385

Table S7 Statistical Evaluation of Model Discrimination

Model	Training cohort		Internal testing cohort		External testing cohort	
	AUC (95% CI)		AUC (95% CI)		AUC (95% CI)	
Model 1	0.811 (0.770~0.852)		0.806 (0.731~0.882)		0.789 (0.721~0.858)	
Model 2	0.840 (0.802~0.877)		0.835 (0.768~0.901)		0.794 (0.729~0.859)	
Model 3	0.841 (0.803~0.879)		0.811 (0.741~0.881)		0.757 (0.688~0.826)	
Model 4	0.891 (0.862~0.921)		0.884 (0.828~0.940)		0.848 (0.791~0.904)	
Model 5	0.894 (0.864~0.923)		0.860 (0.799~0.920)		0.802 (0.739~0.864)	
	**DeLong (95% CI)**	***p***	**DeLong (95% CI)**	***p***	**DeLong (95% CI)**	***p***
Model4 vs. Model1	0.080 (0.037~0.124)	**< 0.001**	0.078 (-0.009~0.164)	0.079	0.059 (-0.022~0.139)	0.153
Model4 vs. Model2	0.051 (0.029~0.074)	**< 0.001**	0.049 (0.018~0.080)	**0.002**	0.054 (0.010~0.098)	**0.016**
Model4 vs. Model3	0.050 (0.023~0.077)	**< 0.001**	0.073 (0.033~0.113)	**< 0.001**	0.091 (0.040~0.142)	**< 0.001**
Model4 vs. Model5	0.002 (-0.013~0.008)	0.665	0.024 (-0.002~0.050)	0.071	0.046 (0.012~0.081)	**0.009**

CI = confidence interval.

Statistically significant p values are bold.

Table S8 Univariable and Multivariable Logistic Regression Analyses of Categorized Radscore and Clinical Variables

**Variables（Radscore as category）**	**Univariable logistic regression analysis**			**Multivariable logistic regression analysis**		
	**OR**	**95% CI**	***p***	**OR**	**95% CI**	***p***
Model 4						
Gender (Ref: female)	1.257	0.832~1.902	0.28			
Age*	1.09	1.070~1.113	**< 0.001**	1.081	1.059~1.105	**< 0.001**
Location (Ref: lower) *	1.892	1.245~2.891	**0.003**	1.99	1.154~3.469	**0.01**
Radscore (Ref: lower) *	10.518	6.667~16.942	**< 0.001**	8.106	4.877~13.793	**< 0.001**
Model5						
Gender (Ref: female)	1.257	0.832~1.902	0.28			
Age*	1.09	1.070~1.113	**< 0.001**	1.081	1.059~1.106	**< 0.001**
Location (Ref: lower) *	1.892	1.245~2.891	**0.003**	1.881	1.092~3.269	**0.02**
Radscore (Ref: lower) *	10.585	6.724 ~ 17.000	**< 0.001**	8.109	4.893~13.747	**< 0.001**

**Variables（Radscore as category）**	**Coef**	**OR**	**95% CI**	***p***
Model 4				
Age	0.078	1.081	1.059~1.105	**< 0.001**
Location (Ref: lower)	0.688	1.99	1.154~3.469	**0.01**
Radscore (Ref: lower)	2.093	8.106	4.877~13.793	**< 0.001**
Intercept	-5.921	0		**< 0.001**
Model 5				
Age	0.078	1.081	1.059~1.106	**< 0.001**
Location (Ref: lower)	0.632	1.881	1.092~3.269	**0.02**
Radscore (Ref: lower)	2.093	8.109	4.893~13.747	**< 0.001**
Intercept	-5.951	0		**< 0.001**

Statistically significant p values are bold*

Variables with p < 0.2 on univariable analysis were included in multivariable analysis

OR = odds ratio, CI = confidence interval.

Table S9 Predictive Performance of Models Using Categorical Radscore Derived from Model 4 and Model 5

Model	Training cohort (n = 407)	Internal validation cohort (n =146)	External validation cohort (n = 185)
Model 4 (Radscore as Category)			
AUC	0.866 (0.832~0.900)	0.827 (0.760~0.893)	0.823 (0.763~0.883)
Sensitivity	0.853 (0.750~0.902)	0.847 (0.800~0.953)	0.856 (0.722~0.948)
Specificity	0.729 (0.571~0.797)	0.541 (0.328~0.951)	0.602 (0.483~0.744)
Accuracy	0.791	0.719	0.735
Precision	0.760	0.720	0.703
F1 score	0.804	0.778	0.772
Cutoff		-0.380	
Model 5 (Radscore as Category)			
AUC	0.867 (0.833~0.901)	0.816 (0.748~0.885)	0.773 (0.706~0.840)
Sensitivity	0.725 (0.602~0.789)	0.753 (0.647~0.871)	0.680 (0.515~0.794)
Specificity	0.867 (0.752~0.906)	0.721 (0.541~0.902)	0.727 (0.568~0.841)
Accuracy	0.796	0.740	0.703
Precision	0.846	0.790	0.733
F1 score	0.781	0.771	0.706
Cutoff		0.482	

Table S10 Diagnostic Performance Metrics of Individual Radiologists and Consensus Interpretation

Radiologist	Sensitivity	Specificity	Accuracy	Precision	F1 score
Radiologist1	0.791	0.819	0.804	0.842	0.816
Radiologist2	0.775	0.805	0.789	0.829	0.801
Radiologist3	0.725	0.852	0.782	0.857	0.786
Radiologist4	0.703	0.718	0.71	0.753	0.727
Radiologist5	0.659	0.765	0.707	0.774	0.712
Radiologist6	0.626	0.758	0.686	0.76	0.687
Consensus of radiologists	0.813	0.779	0.798	0.818	0.815

Table S11 Subgroup Analysis of Model Performance

	AUC	Sensitivity	Specificity	Accuracy	Precision	F1 score
Age						
≥ 65	0.866 (0.831~0.900)	0.718 (0.624~0.788)	0.856 (0.763~0.910)	0.804	0.753	0.735
< 65	0.822 (0.771~0.874)	0.907 (0.847~0.954)	0.496 (0.284~0.649)	0.800	0.838	0.871
Sex						
Female	0.862 (0.817~0.906)	0.697 (0.513~0.807)	0.880 (0.789~0.932)	0.794	0.838	0.761
Male	0.890 (0.863~0.918)	0.880 (0.828~0.921)	0.717 (0.575~0.799)	0.807	0.791	0.833
Radiologist						
Senior	0.900 (0.869~0.931)	0.835 (0.736~0.890)	0.807 (0.665~0.876)	0.822	0.831	0.833
Junior	0.859 (0.823~0.895)	0.818 (0.749~0.887)	0.754 (0.644~0.843)	0.787	0.779	0.798
Location						
Upper/middle lobe	0.879 (0.848~0.909)	0.870 (0.795~0.913)	0.752 (0.626~0.824)	0.815	0.801	0.834
Lower lobe	0.883 (0.845~0.922)	0.735 (0.621~0.841)	0.823 (0.723~0.915)	0.779	0.808	0.770
Size						
Large	0.906 (0.880~0.932)	0.905 (0.849~0.944)	0.691 (0.562~0.781)	0.823	0.824	0.863
Small	0.787 (0.731~0.843)	0.594 (0.406~0.703)	0.868 (0.770~0.931)	0.767	0.723	0.652

AUC = area under the receiver-operating-characteristics curve.

Fig. S1 Construction and evaluation of Model 1. (A) Feature importance of Model 1. **(B)** Decision curve analysis for Model 1, illustrating the net clinical benefit across a range of threshold probabilities. **(C)** Calibration curve of Model 1.

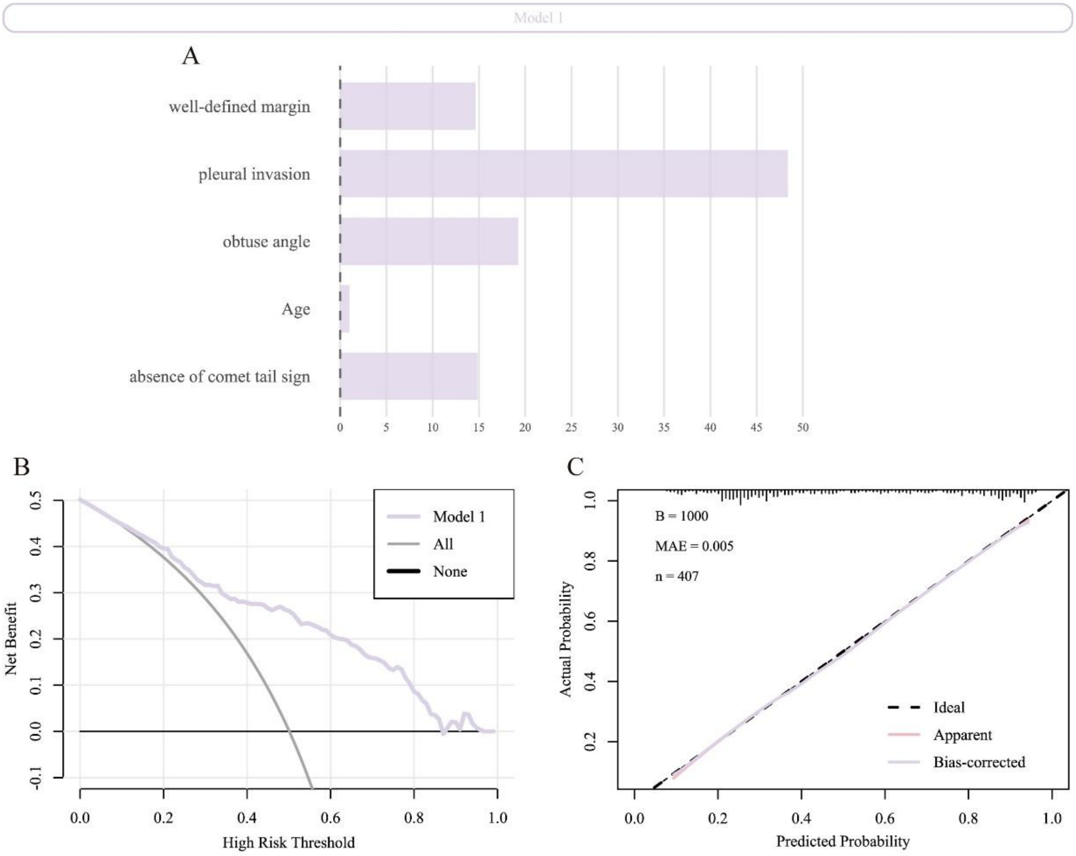

Fig. S2 Inter- and intra-observer reproducibility of radiomic feature extraction. Assessment of inter- and intra-observer reproducibility for radiomic feature extraction using inter-/intra-class correlation coefficients (ICCs). **(A, B)** Inter-observer ICCs, representing the agreement between two independent

readers. **(C, D)** Intra-observer ICCs, reflecting the consistency of feature extraction by the same reader at different time points. Features with both inter- and intra-observer ICCs > 0.75 were considered to exhibit good reproducibility and were retained for further analysis. Features with ICCs < 0.75 were excluded, comprising 138 features with poor inter-observer agreement (46 from the lesion and 92 from the perilesion regions), and 30 features with poor intra-observer consistency (26 from the lesion and 4 from the perilesion regions).

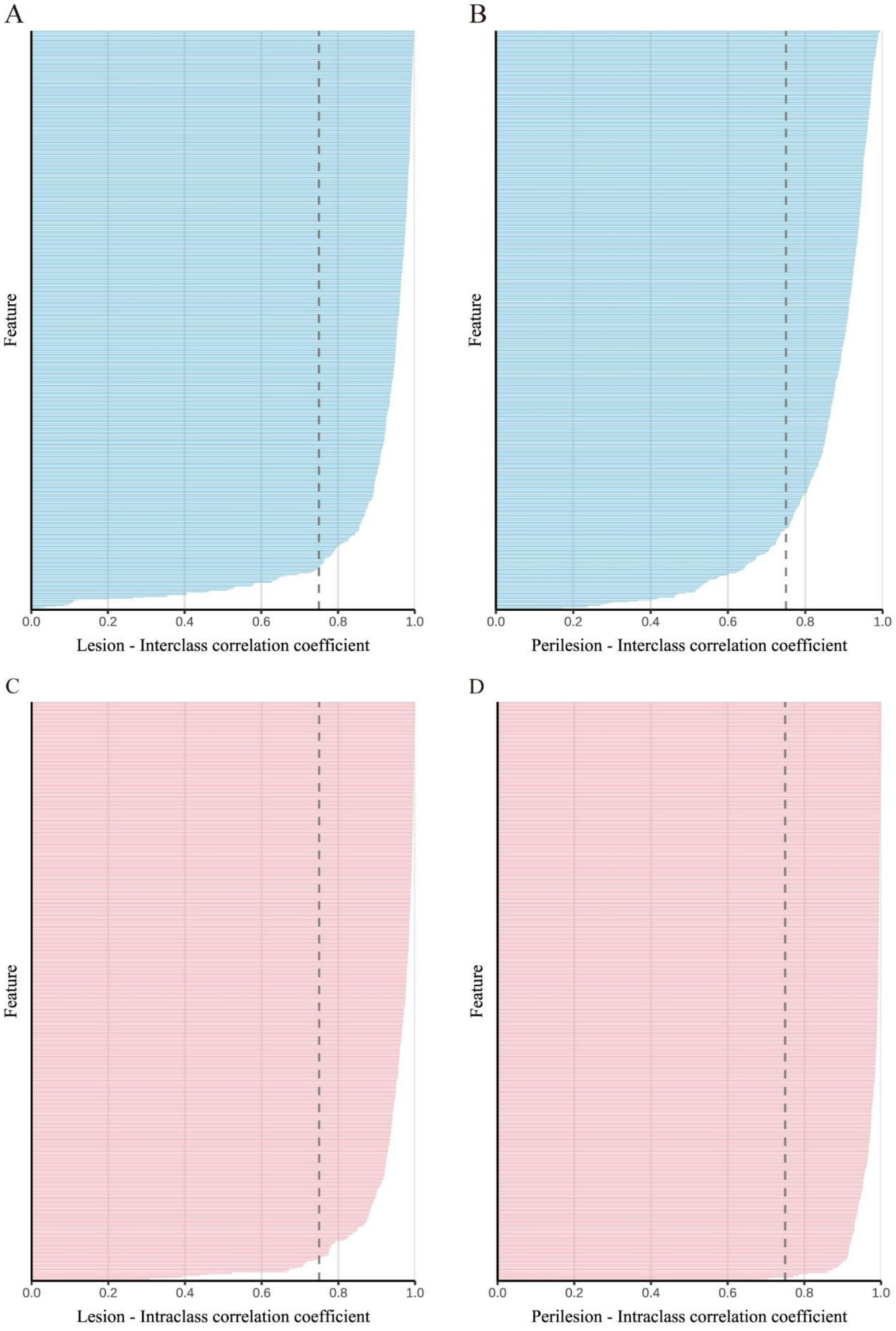
A
B
C
D
Feature
Feature
Feature
Feature
0.0
0.2
0.4
0.6
0.8
1.0
Lesion - Interclass correlation coefficient
Perilesion - Interclass correlation coefficient
Lesion - Intraclass correlation coefficient
Perilesion - Intraclass correlation coefficient

Fig. S3 Pearson correlation analysis of radiomic features. (A) Correlation matrix of features extracted from lesion regions. **(B)** Correlation matrix of features extracted from combined lesion and perilesional regions. Features with Pearson correlation coefficients greater than 0.9 were considered highly correlated and excluded from subsequent modeling.

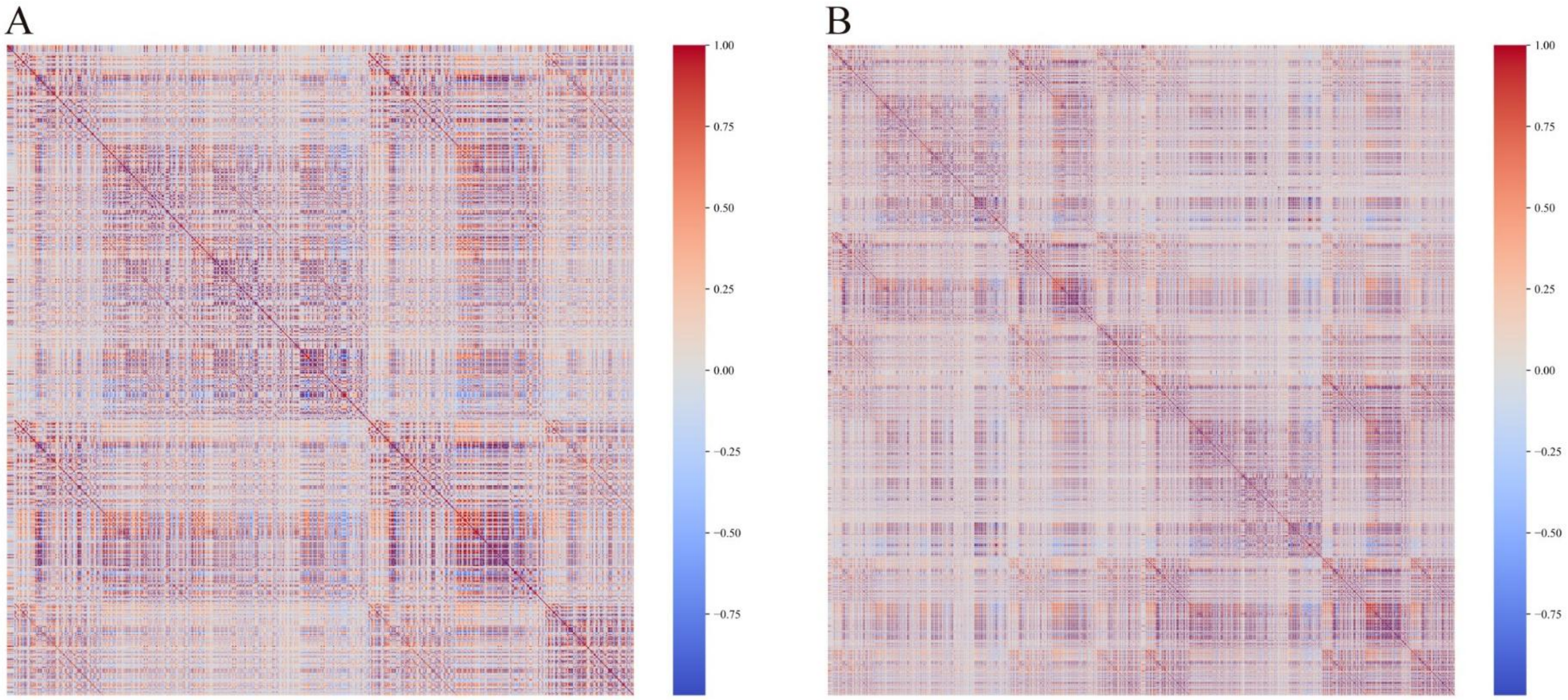

Fig. S4 Construction and evaluation of Model 2. (A) LASSO tuning parameter (λ) selection via 10-fold cross-validation. The optimal λ was chosen using the λ_{1se} criterion. **(B)** LASSO coefficient profiles of 660 radiomic features, with 23 features retaining nonzero coefficients at the selected λ. **(C)** Heatmap of pearson correlation coefficients among final selected radiomic features. **(D)** Bar plot of coefficients for features included in the final model. **(E)** Decision curve analysis for Model 2, illustrating the net clinical benefit across a range of threshold probabilities. **(F)** Calibration curve assessing agreement between predicted and observed outcomes.

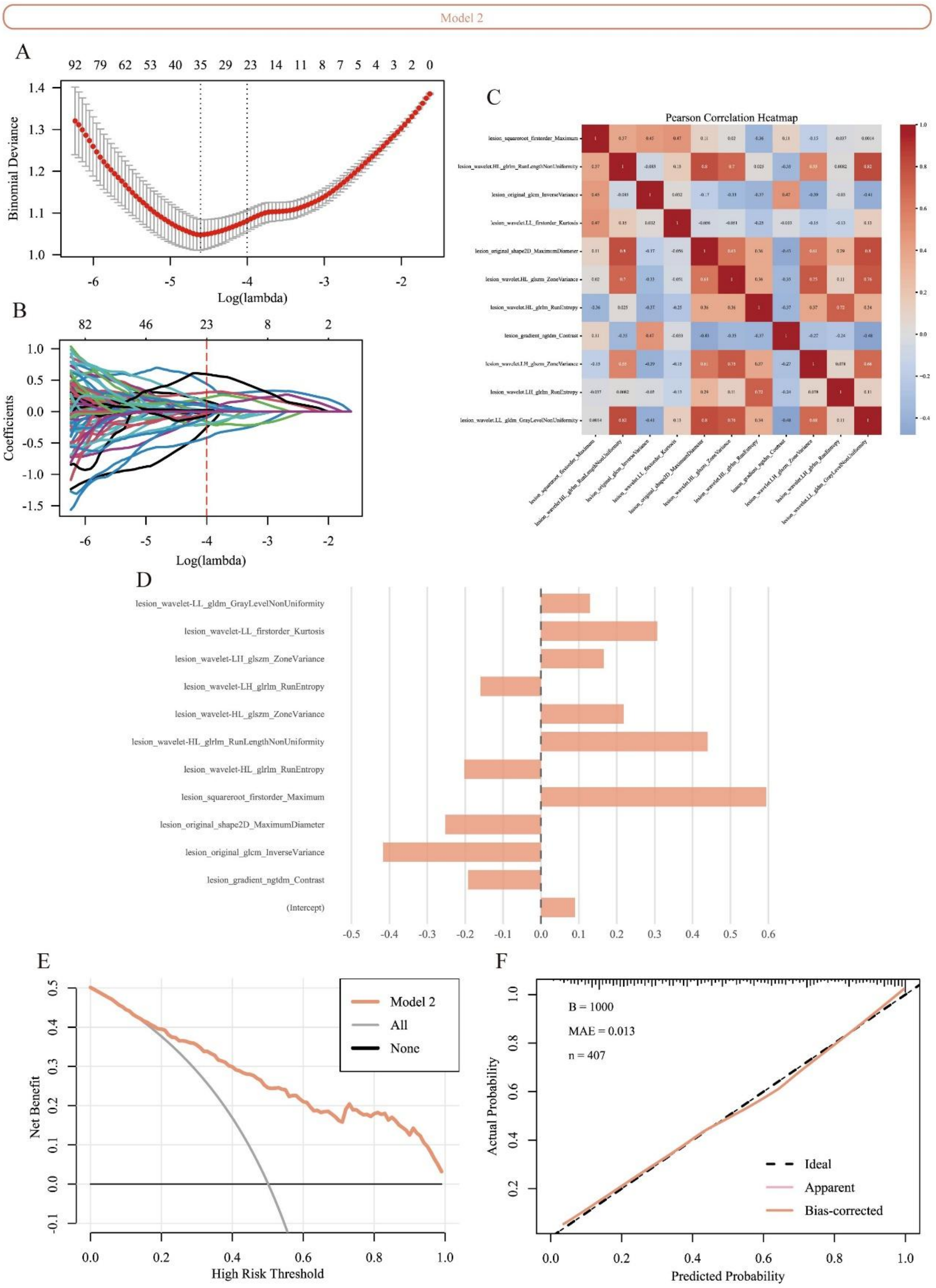
Model 2
A
Binomial Deviance
Log(lambda)
B
Coefficients
Log(lambda)
C
Pearson Correlation Heatmap
D
lesion_wavelet-LL_gldm_GrayLevelNonUniformity
lesion_wavelet-LL_firstorder_Kurtosis
lesion_wavelet-LH_glszm_ZoneVariance
lesion_wavelet-LH_glrlm_RunEntropy
lesion_wavelet-HL_glszm_ZoneVariance
lesion_wavelet-HL_glrlm_RunLengthNonUniformity
lesion_wavelet-HL_glrlm_RunEntropy
lesion_squareroot_firstorder_Maximum
lesion_original_shape2D_MaximumDiameter
lesion_original_glcm_InverseVariance
lesion_gradient_ngtdm_Contrast
(Intercept)
E
Model 2
All
None
Net Benefit
High Risk Threshold
F
B = 1000
MAE = 0.013
n = 407
Ideal
Apparent
Bias-corrected
Actual Probability
Predicted Probability

Fig. S5 Construction and evaluation of Model 3. (A) LASSO tuning parameter (λ) selection via 10-fold cross-validation. The optimal λ was chosen using the λ_{1se} criterion. **(B)** LASSO coefficient profiles of 1320 radiomic features, with 22 features retaining nonzero coefficients at the selected λ. **(C)** Heatmap of pearson correlation coefficients among final selected radiomic features. **(D)** Bar plot of coefficients for features included in the final model. **(E)** Decision curve analysis for Model 3, illustrating the net clinical benefit across a range of threshold probabilities. **(F)** Calibration curve assessing agreement between predicted and observed outcomes.

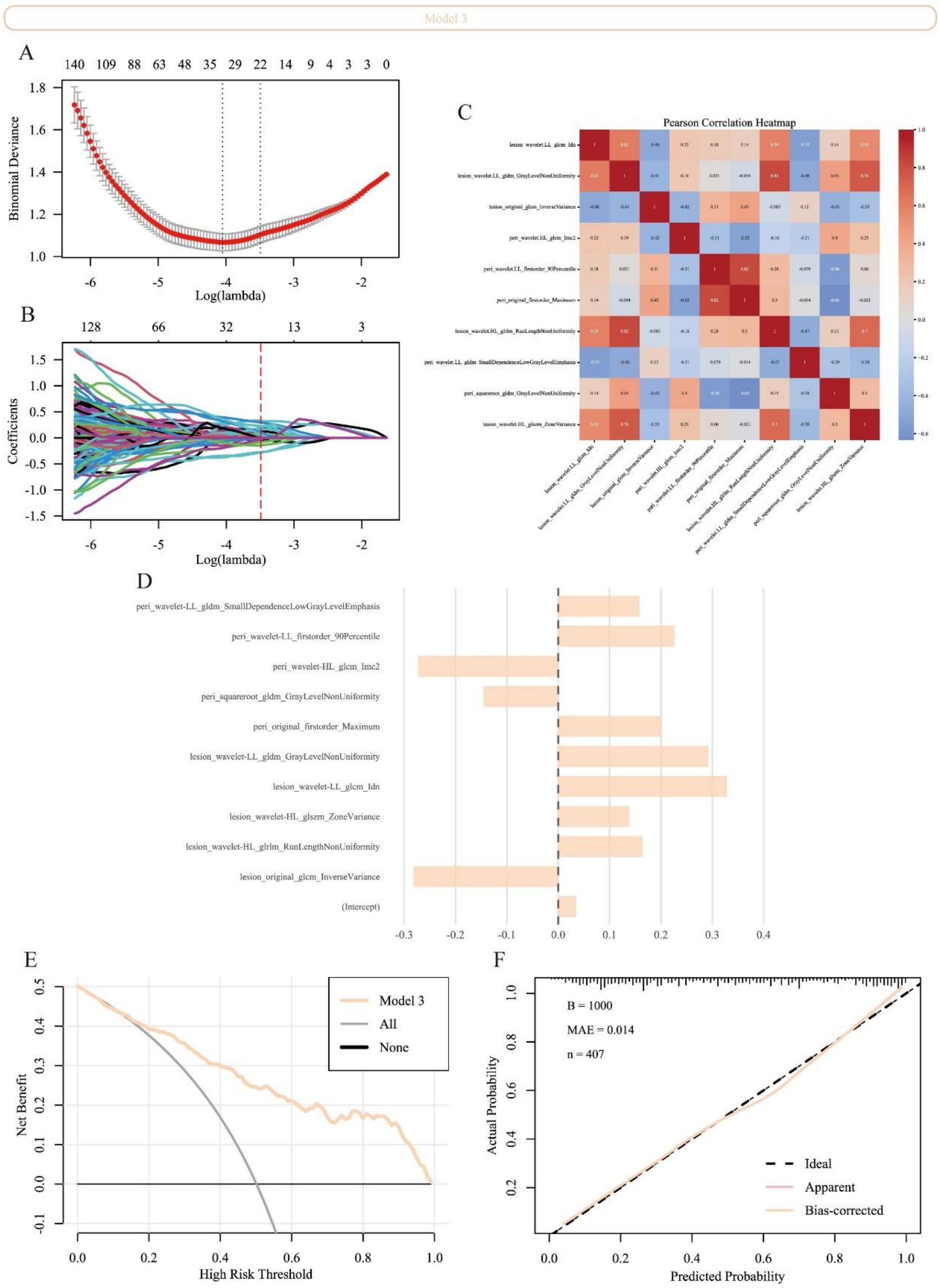
Model 3
A
Binomial Deviance
Log(lambda)
B
Coefficients
Log(lambda)
C
Pearson Correlation Heatmap
D
peri_wavelet-LL_gldm_SmallDependenceLowGrayLevelEmphasis
peri_wavelet-LL_firstorder_90Percentile
peri_wavelet-HL_glcm_Imc2
peri_squareroot_gldm_GrayLevelNonUniformity
peri_original_firstorder_Maximum
lesion_wavelet-LL_gldm_GrayLevelNonUniformity
lesion_wavelet-LL_glcm_Idn
lesion_wavelet-HL_glszm_ZoneVariance
lesion_wavelet-HL_glrlm_RunLengthNonUniformity
lesion_original_glcm_InverseVariance
(Intercept)
E
Model 3
All
None
Net Benefit
High Risk Threshold
F
B = 1000
MAE = 0.014
n = 407
Ideal
Apparent
Bias-corrected
Actual Probability
Predicted Probability

Fig. S6 Construction and evaluation of Model 5. (A) The nomogram of Model 5, constructed based on Radscore and clinical features. **(B)** Relative contribution of different feature classes in the Model 5. **(C)** Decision curve analysis for Model 5, illustrating the net clinical benefit across a range of threshold probabilities. **(D)** Calibration curve assessing agreement between predicted and observed outcomes.

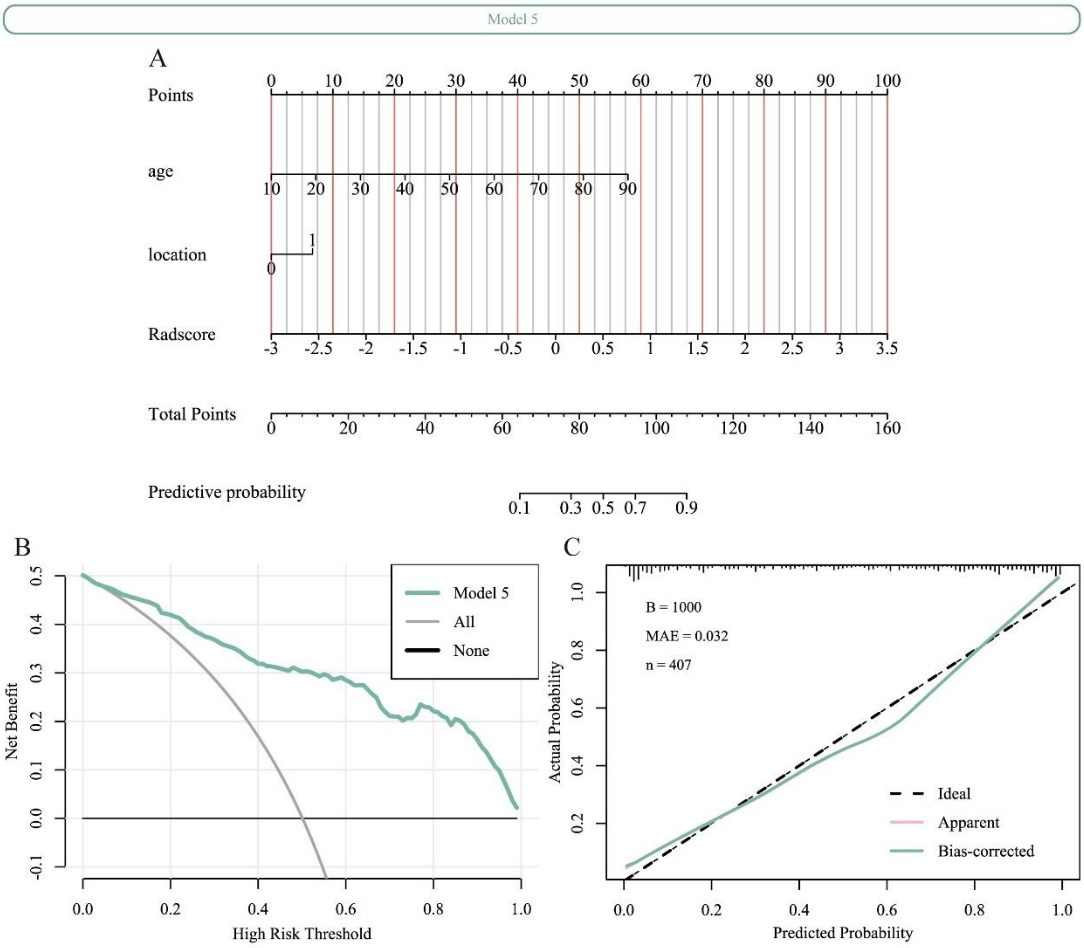

Fig. S7 Comparative diagnostic performance of five models in the training cohort. Confusion matrices displaying classification results of the five models. Radar plots summarizing key diagnostic metrics (accuracy, sensitivity, specificity, precision, F1-score) of five models. Beeswarm plots showing the distribution of model-predicted scores between benign and malignant SPLs, with all models demonstrating statistically significant differences between the two groups. SPLs, subpleural pulmonary lesions.

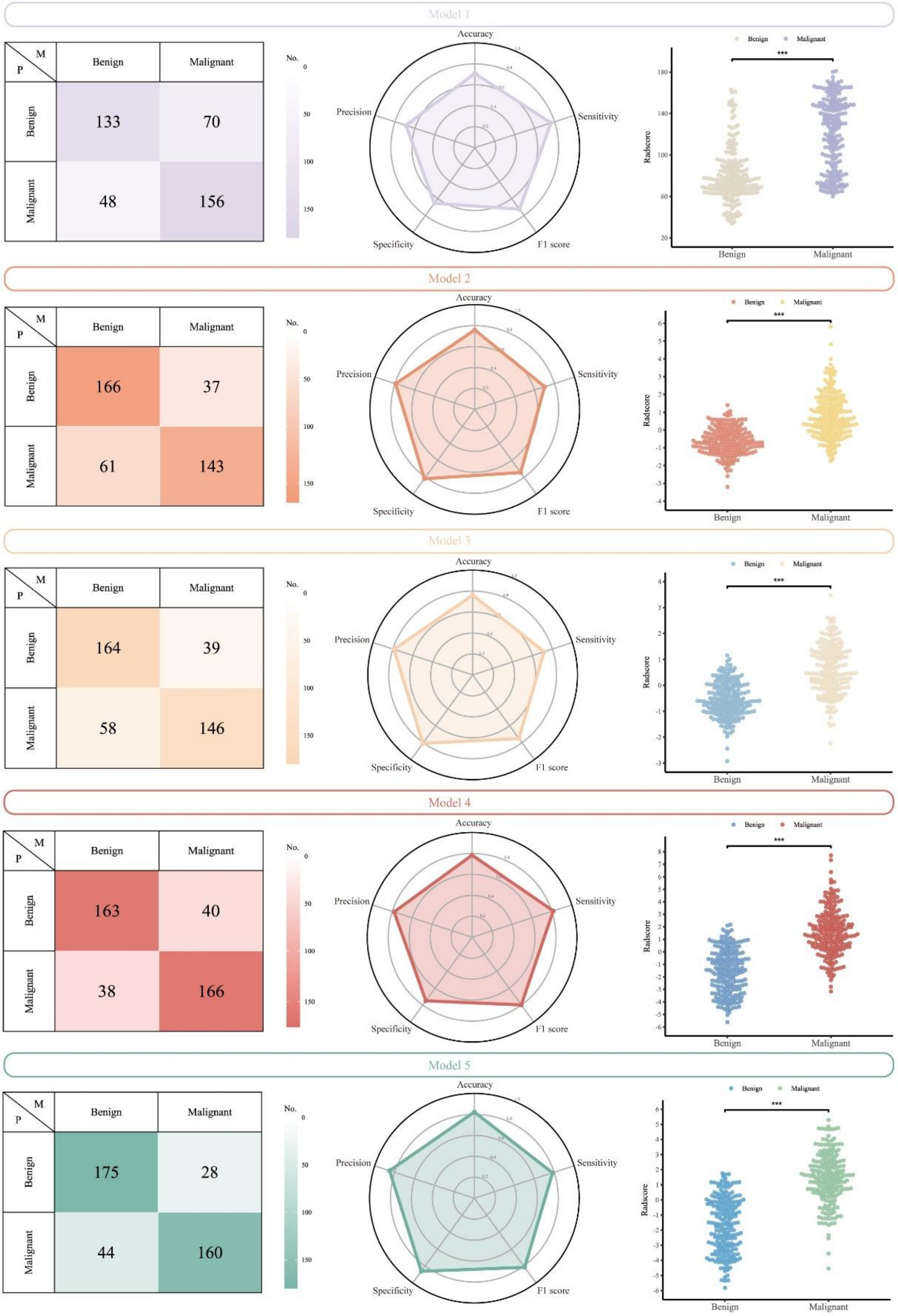
Model 1
M
P
Benign
Malignant
Benign
Malignant
133
70
48
156
No.
0
50
100
150
Accuracy
Sensitivity
F1 score
Specificity
Precision
Benign
Malignant
***
Radscore
Benign
Malignant
Model 2
M
P
Benign
Malignant
Benign
Malignant
166
37
61
143
No.
0
50
100
150
Accuracy
Sensitivity
F1 score
Specificity
Precision
Benign
Malignant
***
Radscore
Benign
Malignant
Model 3
M
P
Benign
Malignant
Benign
Malignant
164
39
58
146
No.
0
50
100
150
Accuracy
Sensitivity
F1 score
Specificity
Precision
Benign
Malignant
***
Radscore
Benign
Malignant
Model 4
M
P
Benign
Malignant
Benign
Malignant
163
40
38
166
No.
0
50
100
150
Accuracy
Sensitivity
F1 score
Specificity
Precision
Benign
Malignant
***
Radscore
Benign
Malignant
Model 5
M
P
Benign
Malignant
Benign
Malignant
175
28
44
160
No.
0
50
100
150
Accuracy
Sensitivity
F1 score
Specificity
Precision
Benign
Malignant
***
Radscore
Benign
Malignant

Fig. S8 Comparative diagnostic performance of five models in the internal validation cohort.

Confusion matrices displaying classification results of the five models. Radar plots summarizing key diagnostic metrics (accuracy, sensitivity, specificity, precision, F1-score) of five models. Beeswarm plots showing the distribution of model-predicted scores between benign and malignant SPLs, with all models demonstrating statistically significant differences between the two groups. SPLs, subpleural pulmonary lesions.

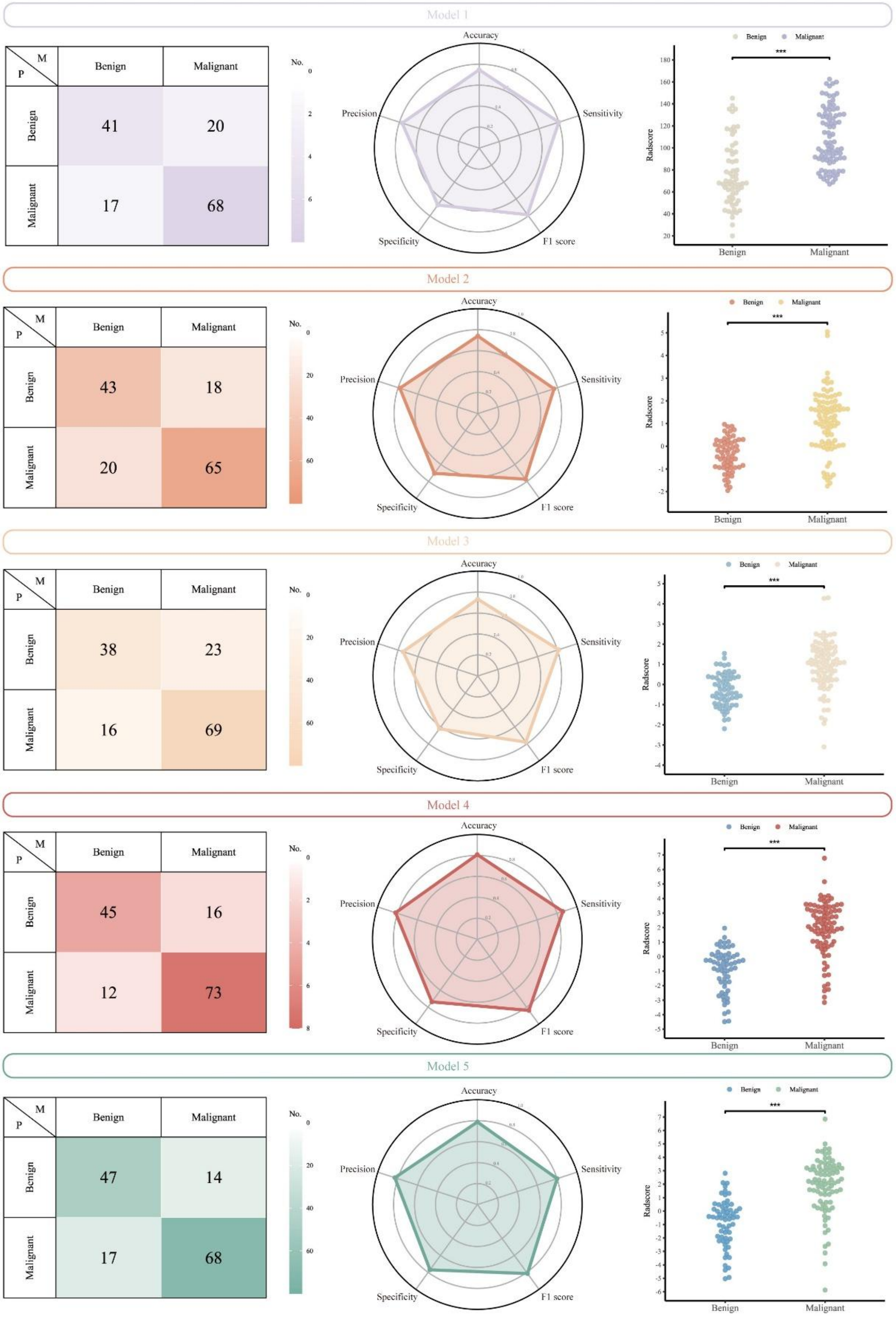
Model 1
M
P
Benign
Malignant
41
20
17
68
No.
Accuracy
Precision
Sensitivity
Specificity
F1 score
Radscore
***
Model 2
M
P
Benign
Malignant
43
18
20
65
No.
Accuracy
Precision
Sensitivity
Specificity
F1 score
Radscore
***
Model 3
M
P
Benign
Malignant
38
23
16
69
No.
Accuracy
Precision
Sensitivity
Specificity
F1 score
Radscore
***
Model 4
M
P
Benign
Malignant
45
16
12
73
No.
Accuracy
Precision
Sensitivity
Specificity
F1 score
Radscore
***
Model 5
M
P
Benign
Malignant
47
14
17
68
No.
Accuracy
Precision
Sensitivity
Specificity
F1 score
Radscore
***

Fig. S9 Decision and calibration curve analysis of five models in the internal validation cohort.

(A) Decision curve analysis of five models, showing net clinical benefit across a range of threshold probabilities. **(B)** Calibration curves of five models, demonstrating agreement between predicted and observed outcomes in the internal validation cohort.

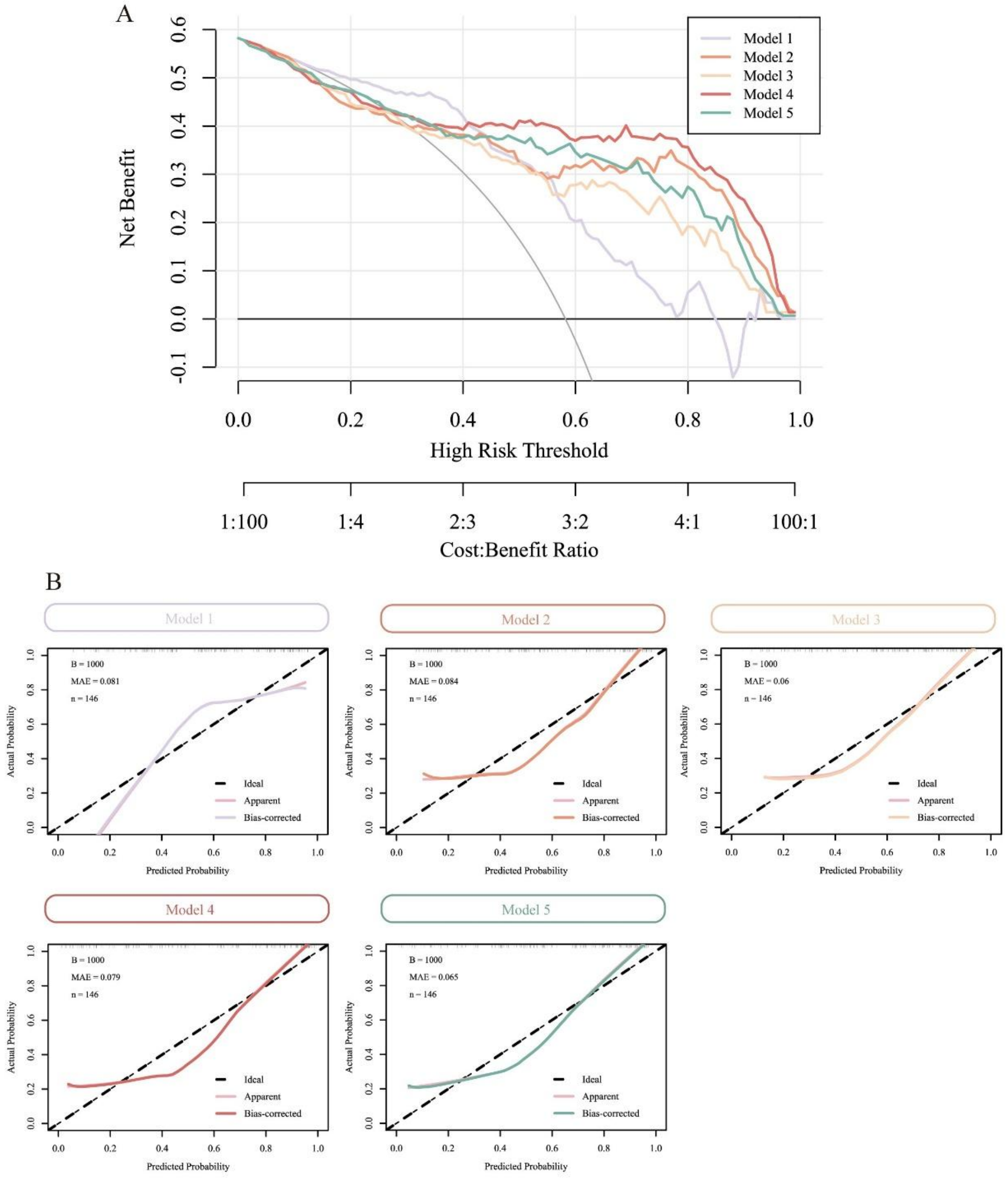

Fig. S10 Decision and calibration curve analysis of five models in the external validation cohort.

(A) Decision curve analysis of five models, showing net clinical benefit across a range of threshold probabilities. **(B)** Calibration curves of five models, demonstrating agreement between predicted and observed outcomes in the external validation cohort.

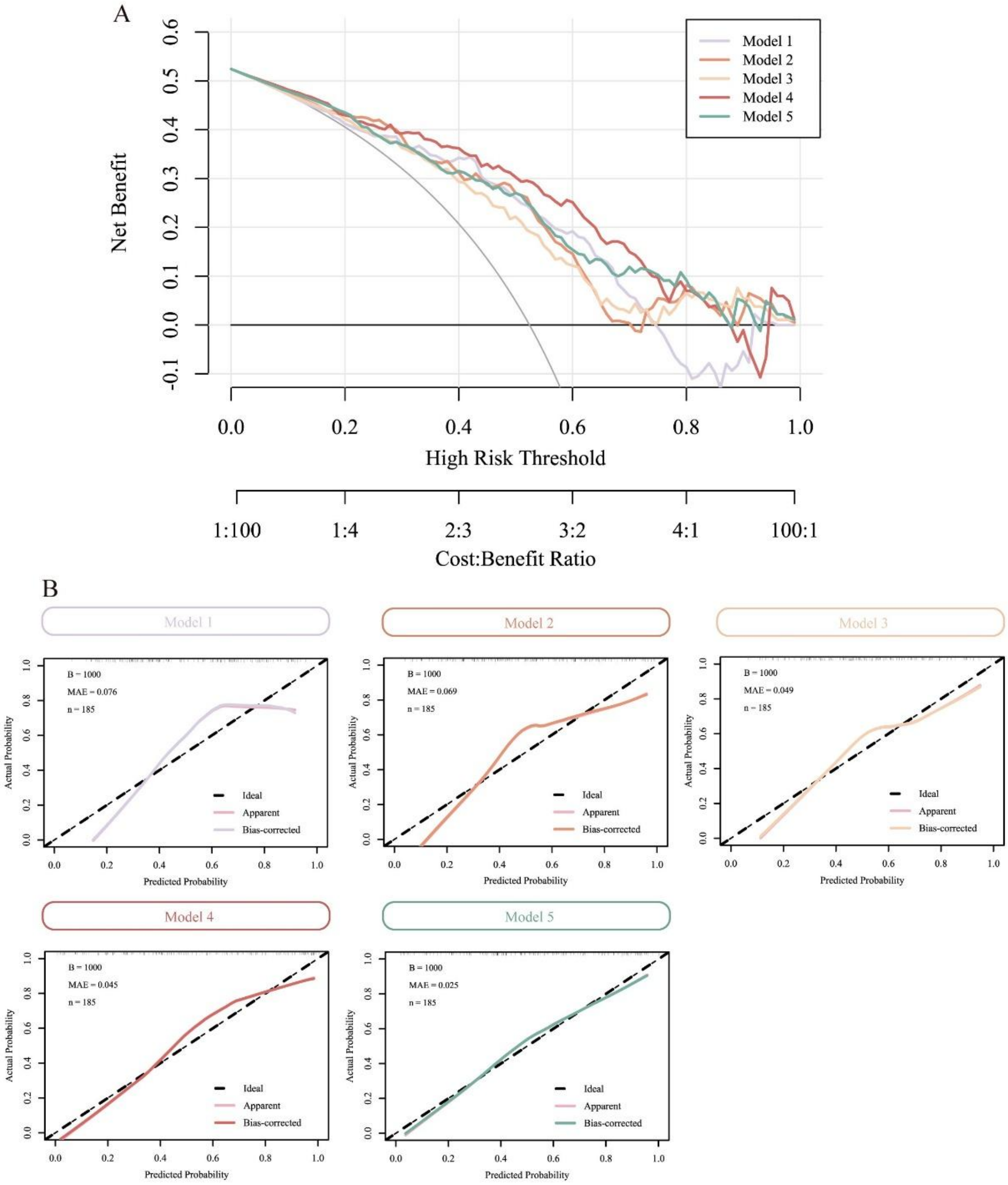

Fig. S11 Construction and evaluation of the Binary-Radscore Model 4. (A) Nomogram based on the binary-Radscore, converted from the continuous Radscore of Model 4 using an optimal cutoff. **(B)** Confusion matrix showing classification results in the training cohort. **(C)** Radar plot summarizing key diagnostic metrics (accuracy, sensitivity, specificity, precision, F1-score) of the binary-Radscore Model 4. **(D)** Beeswarm plot illustrating the distribution of model-predicted scores between benign and malignant SPLs, showing a significant difference between the two groups. **(E)** Decision curve analysis assessing the clinical utility of the binary-Radscore Model 4 across a range of threshold probabilities. **(F)** Calibration curve evaluating the agreement between predicted probabilities and observed outcomes.

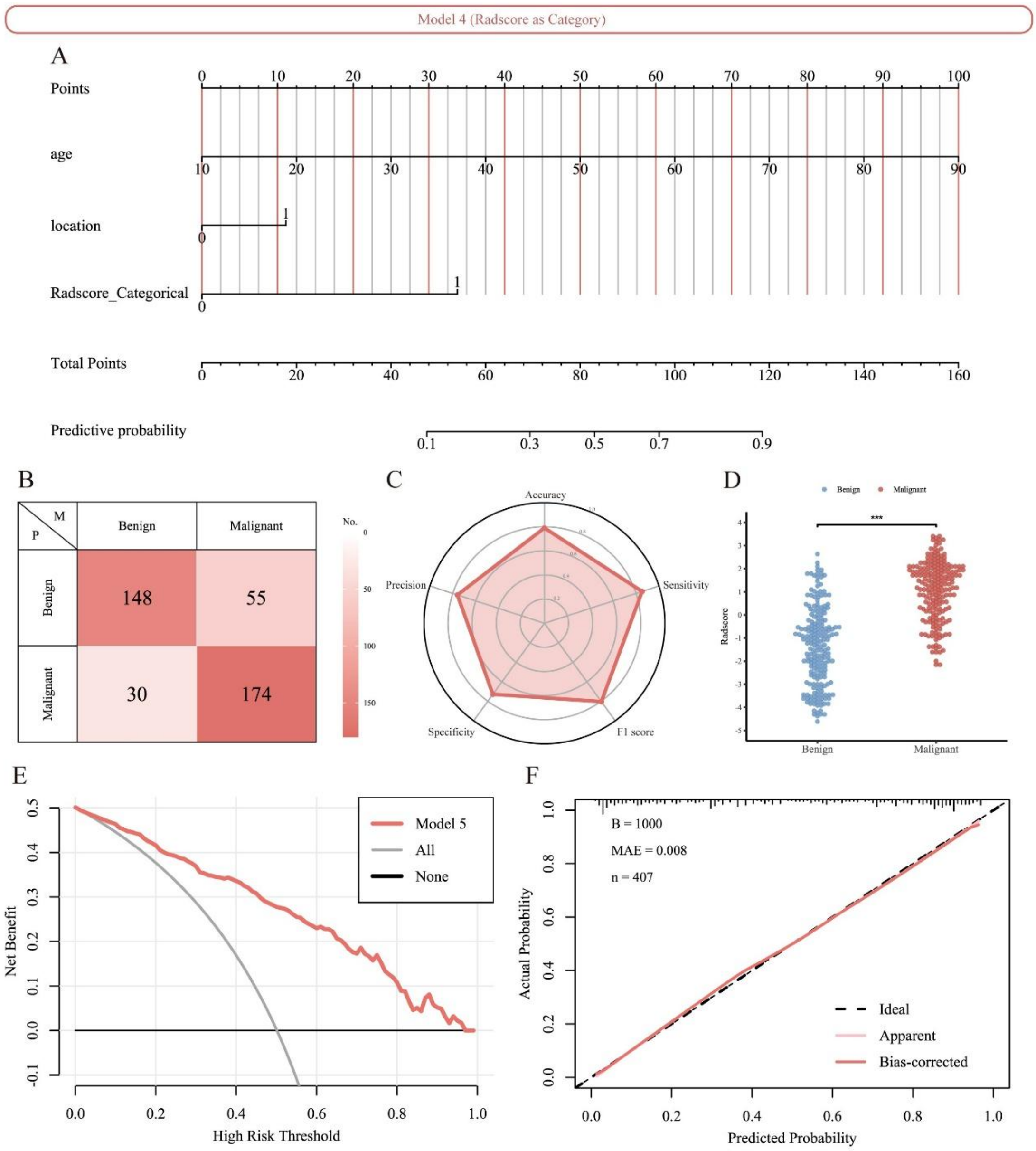

Model 4 (Radscore as Category)
A
Points
age
location
Radscore_Categorical
Total Points
Predictive probability
B
M
P
Benign
Malignant
148
55
30
174
No.
C
Accuracy
Sensitivity
F1 score
Specificity
Precision
D
Benign
Malignant
Radscore
***
E
Model 5
All
None
Net Benefit
High Risk Threshold
F
B = 1000
MAE = 0.008
n = 407
Ideal
Apparent
Bias-corrected
Actual Probability
Predicted Probability

Fig. S12 Construction and evaluation of the Binary-Radscore Model 5. (A) Nomogram based on the binary-Radscore, converted from the continuous Radscore of Model 5 using an optimal cutoff. **(B)** Confusion matrix showing classification results in the training cohort. **(C)** Radar plot summarizing key diagnostic metrics (accuracy, sensitivity, specificity, precision, F1-score) of the binary-Radscore Model 5. **(D)** Beeswarm plot illustrating the distribution of model-predicted scores between benign and malignant SPLs, showing a significant difference between the two groups. **(E)** Decision curve analysis assessing the clinical utility of the binary-Radscore Model 5 across a range of threshold probabilities. **(F)** Calibration curve evaluating the agreement between predicted probabilities and observed outcomes.

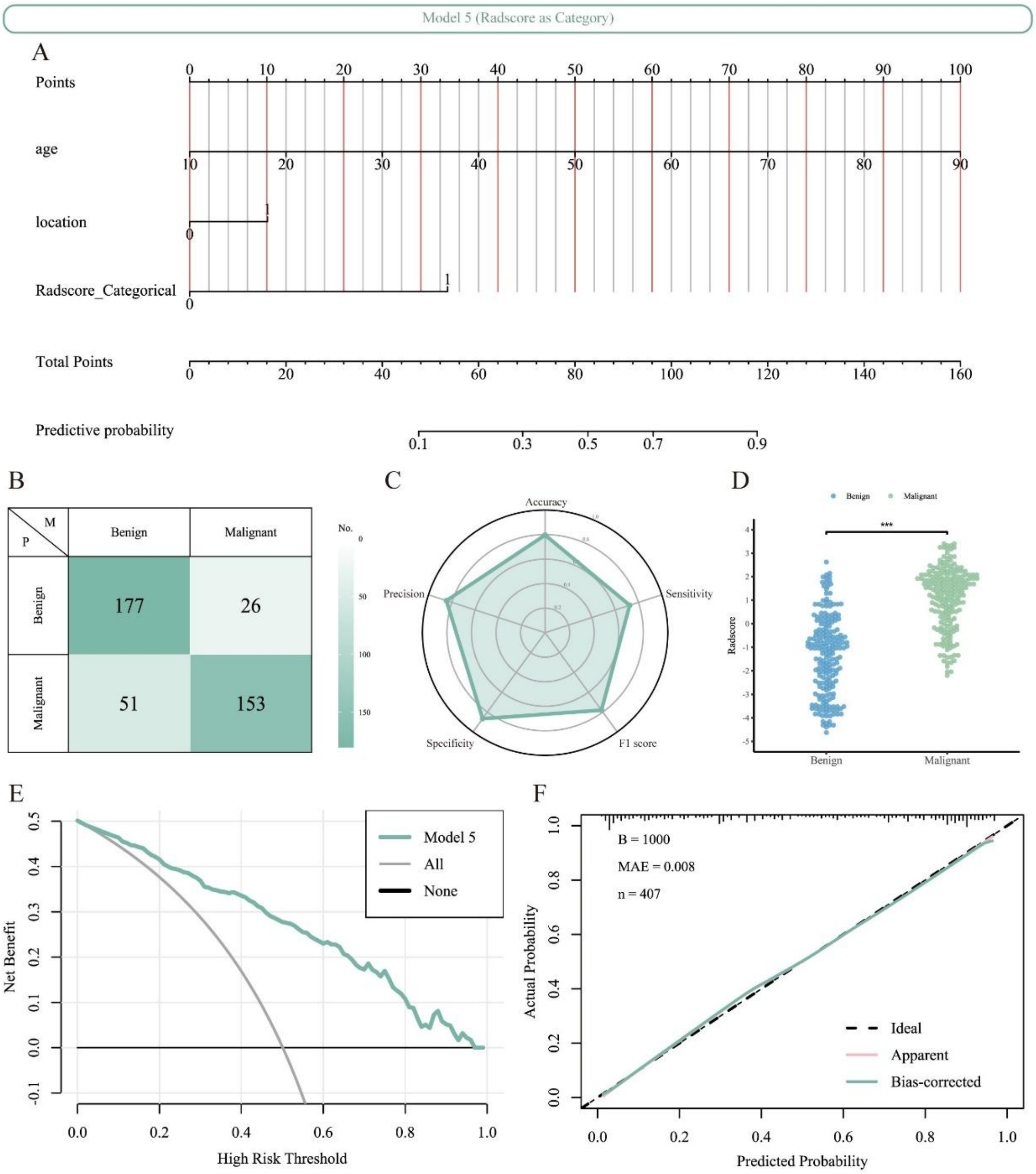
Model 5 (Radscore as Category)
A
Points
age
location
Radscore_Categorical
Total Points
Predictive probability
B
M
P
Benign
Malignant
177
26
51
153
No.
C
Accuracy
Sensitivity
F1 score
Specificity
Precision
D
Benign
Malignant
***
Radscore
E
Model 5
All
None
Net Benefit
High Risk Threshold
F
B = 1000
MAE = 0.008
n = 407
Ideal
Apparent
Bias-corrected
Actual Probability
Predicted Probability

Fig. S13 Diagnostic performance comparison of two Binary-Radscore models (Model 4 and Model 5) in the internal validation cohort. (A, F) Confusion matrices of Binary-Radscore Model 4 and Binary-Radscore Model 5, respectively, showing classification results. (B, G) Radar plots illustrating diagnostic metrics (accuracy, sensitivity, specificity, precision, F1-score) for each model. (C, H) Beeswarm plots displaying the distribution of model-predicted scores between benign and malignant SPLs; both models demonstrate statistically significant differences between the two groups. (D, I) Decision curve analyses demonstrating the clinical net benefit of each model across a range of threshold probabilities. (E, J) Calibration curves evaluating the agreement between predicted probabilities and observed outcomes for Binary-Radscore Model 4 and Binary-Radscore Model 5.

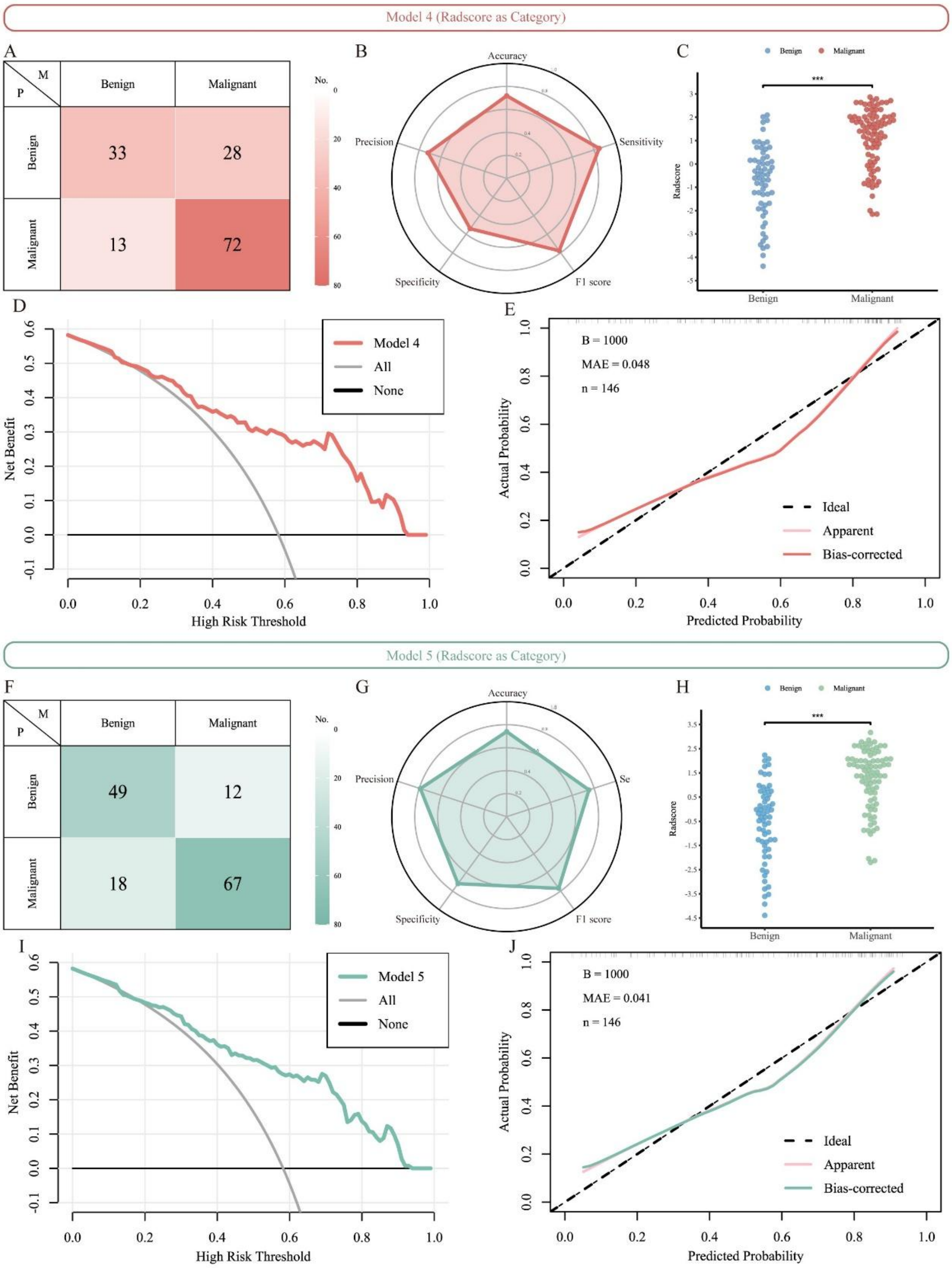
Model 4 (Radscore as Category)
A
M
P
Benign
Malignant
Benign
Malignant
33
28
13
72
No.
B
Accuracy
Sensitivity
F1 score
Specificity
Precision
C
Benign
Malignant
***
Radscore
D
Model 4
All
None
Net Benefit
High Risk Threshold
E
B = 1000
MAE = 0.048
n = 146
Ideal
Apparent
Bias-corrected
Actual Probability
Predicted Probability
Model 5 (Radscore as Category)
F
49
12
18
67
G
H
I
Model 5
J
MAE = 0.041

Fig. S14 Diagnostic performance comparison of two Binary-Radscore models (Model 4 and Model 5) in the external validation cohort. (A, F) Confusion matrices of Binary-Radscore Model 4 and Binary-Radscore Model 5, respectively, showing classification results. (B, G) Radar plots illustrating diagnostic metrics (accuracy, sensitivity, specificity, precision, F1-score) for each model. (C, H) Beeswarm plots displaying the distribution of model-predicted scores between benign and malignant SPLs; both models demonstrate statistically significant differences between the two groups. (D, I) Decision curve analyses demonstrating the clinical net benefit of each model across a range of threshold probabilities. (E, J) Calibration curves evaluating the agreement between predicted probabilities and observed outcomes for Binary-Radscore Model 4 and Binary-Radscore Model 5.

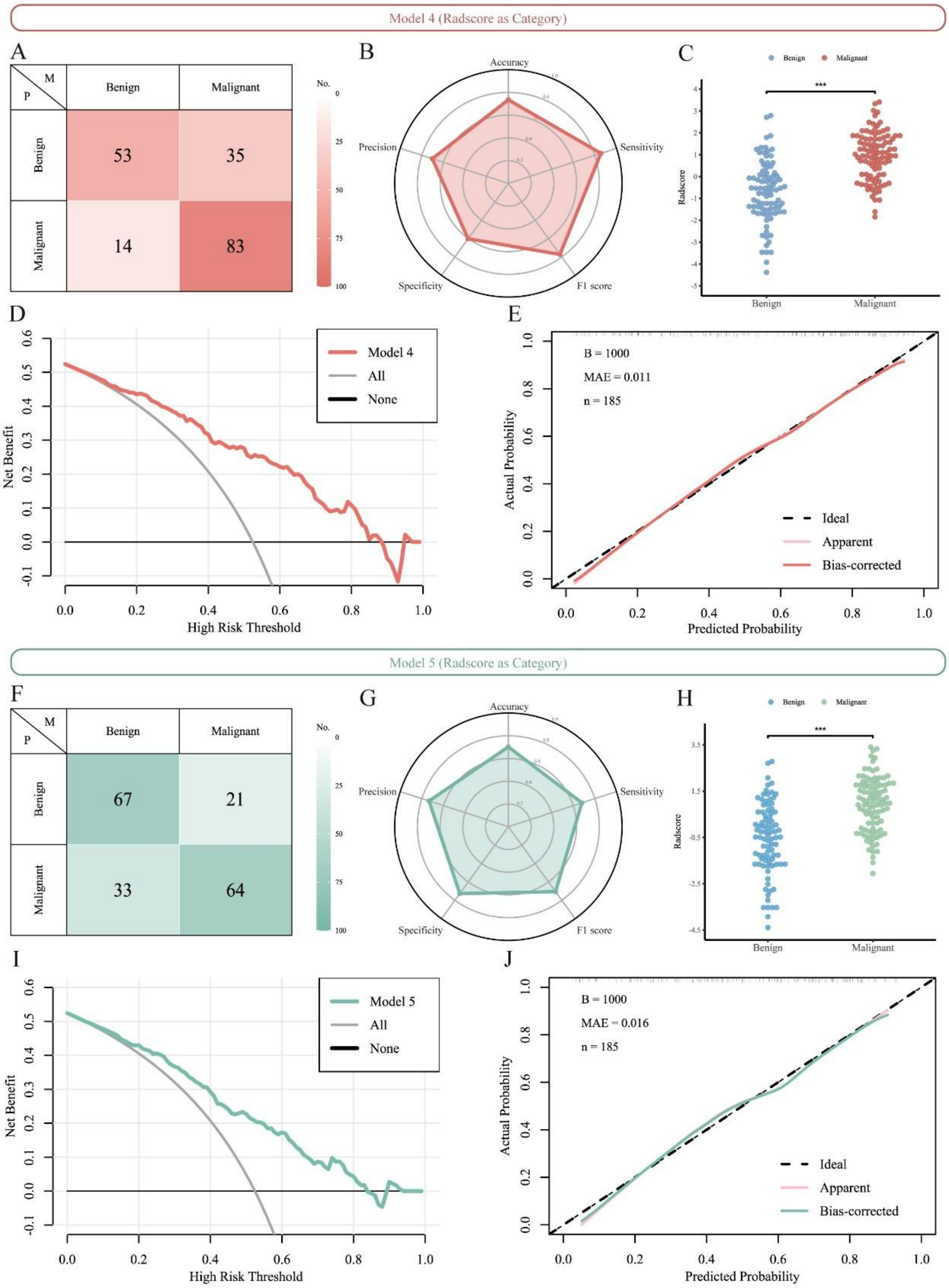
Model 4 (Radscore as Category)
A
M
P
Benign
Malignant
53
35
14
83
No.
0
25
50
75
100
B
Accuracy
Sensitivity
F1 score
Specificity
Precision
C
Benign
Malignant
***
Radscore
D
Model 4
All
None
Net Benefit
High Risk Threshold
E
B = 1000
MAE = 0.011
n = 185
Ideal
Apparent
Bias-corrected
Actual Probability
Predicted Probability
Model 5 (Radscore as Category)
F
M
P
Benign
Malignant
67
21
33
64
No.
0
25
50
75
100
G
Accuracy
Sensitivity
F1 score
Specificity
Precision
H
Benign
Malignant
***
Radscore
I
Model 5
All
None
Net Benefit
High Risk Threshold
J
B = 1000
MAE = 0.016
n = 185
Ideal
Apparent
Bias-corrected
Actual Probability
Predicted Probability

Fig. S15 Inter-reader agreement among six radiologists on the independent test cohort. Most correlation coefficients were greater than 0.50, suggesting moderate correlation in diagnostic performance. These results reflect a reasonable level of consistency among the radiologists when interpreting the same dataset.

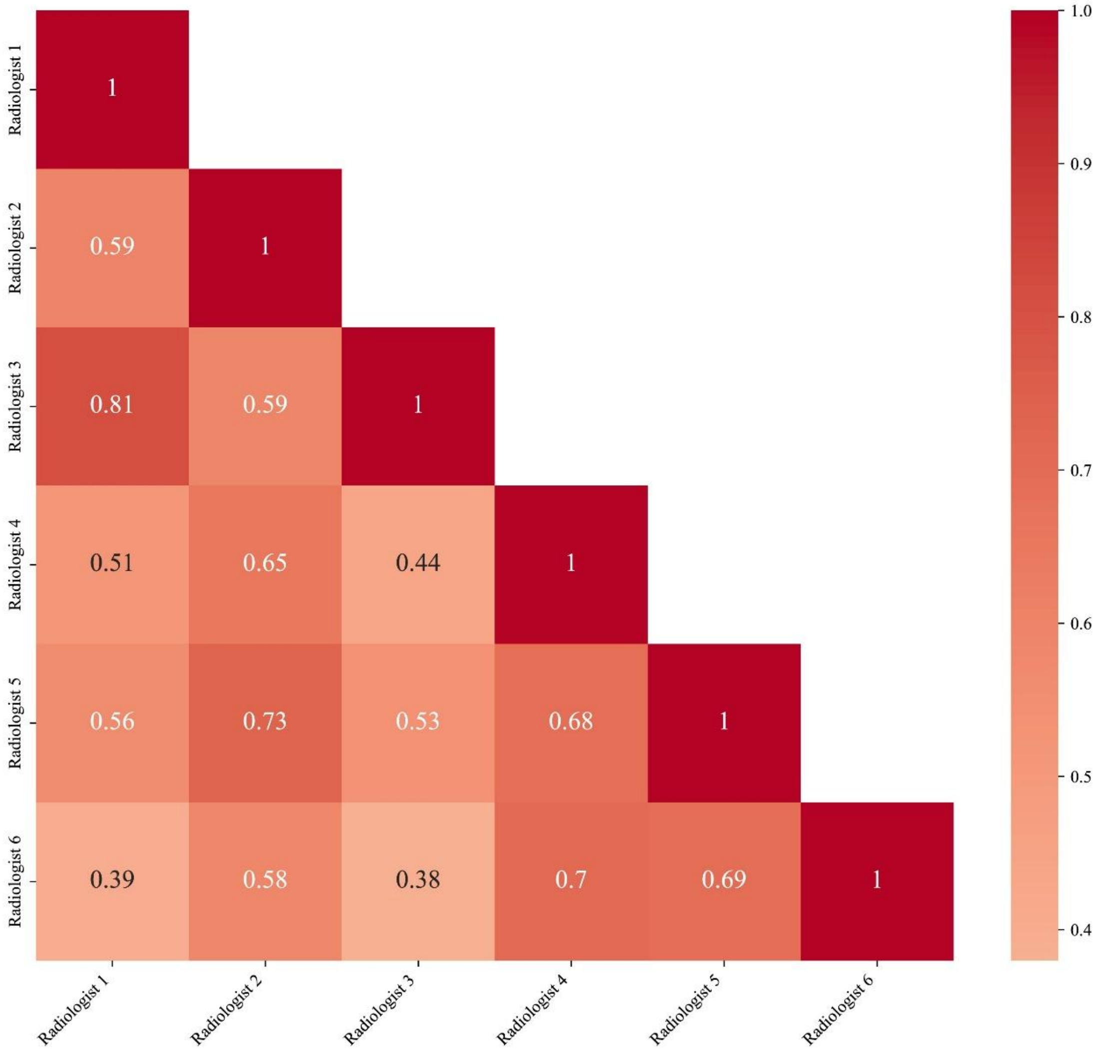

Fig. S16 Diagnostic performance of six radiologists on the independent test cohort. Each radiologist’s performance is visualized through an individual confusion matrix and a corresponding radar plot. The confusion matrices illustrate the classification results for benign and malignant SPLs, while the radar plots summarize key diagnostic metrics (accuracy, sensitivity, specificity, precision, and F1-score), highlighting inter-reader variability in performance.

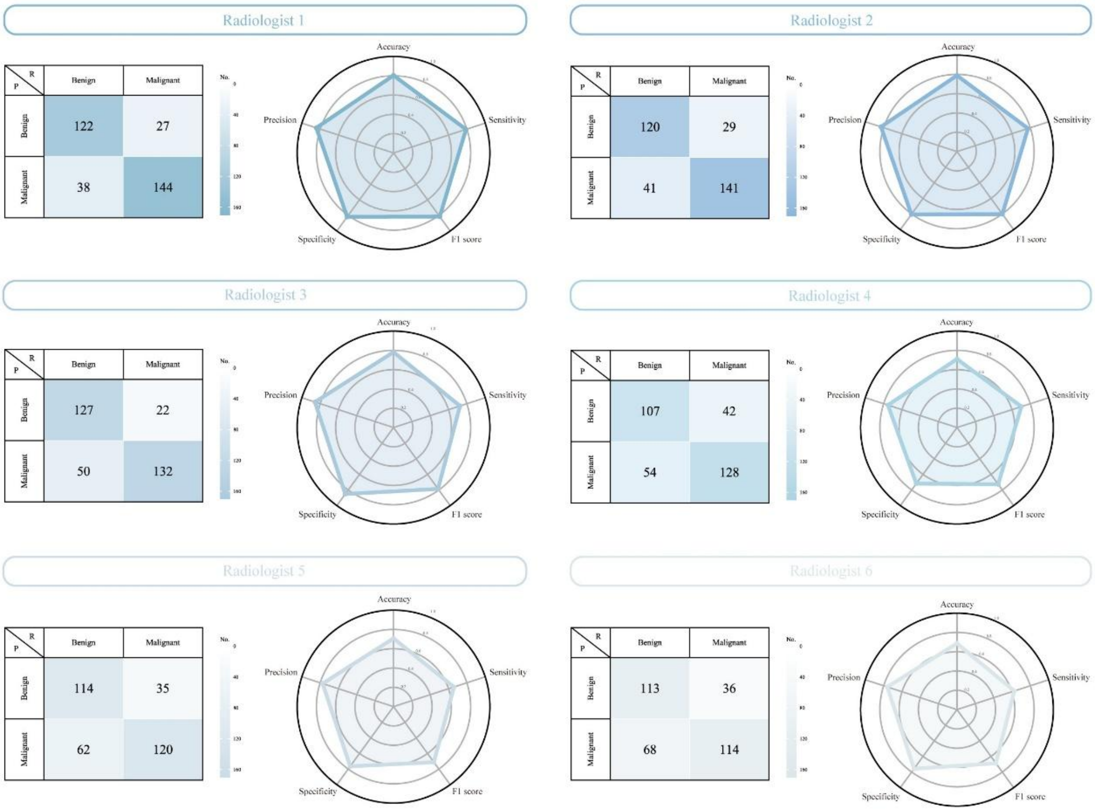

Fig. S17 Diagnostic performance of Model 4 across different subgroups. Confusion matrices illustrate the classification results for each predefined subgroup, while the radar plots summarize key diagnostic metrics (accuracy, sensitivity, specificity, precision, and F1-score) to assess the model's generalizability and robustness across varying clinical scenarios.

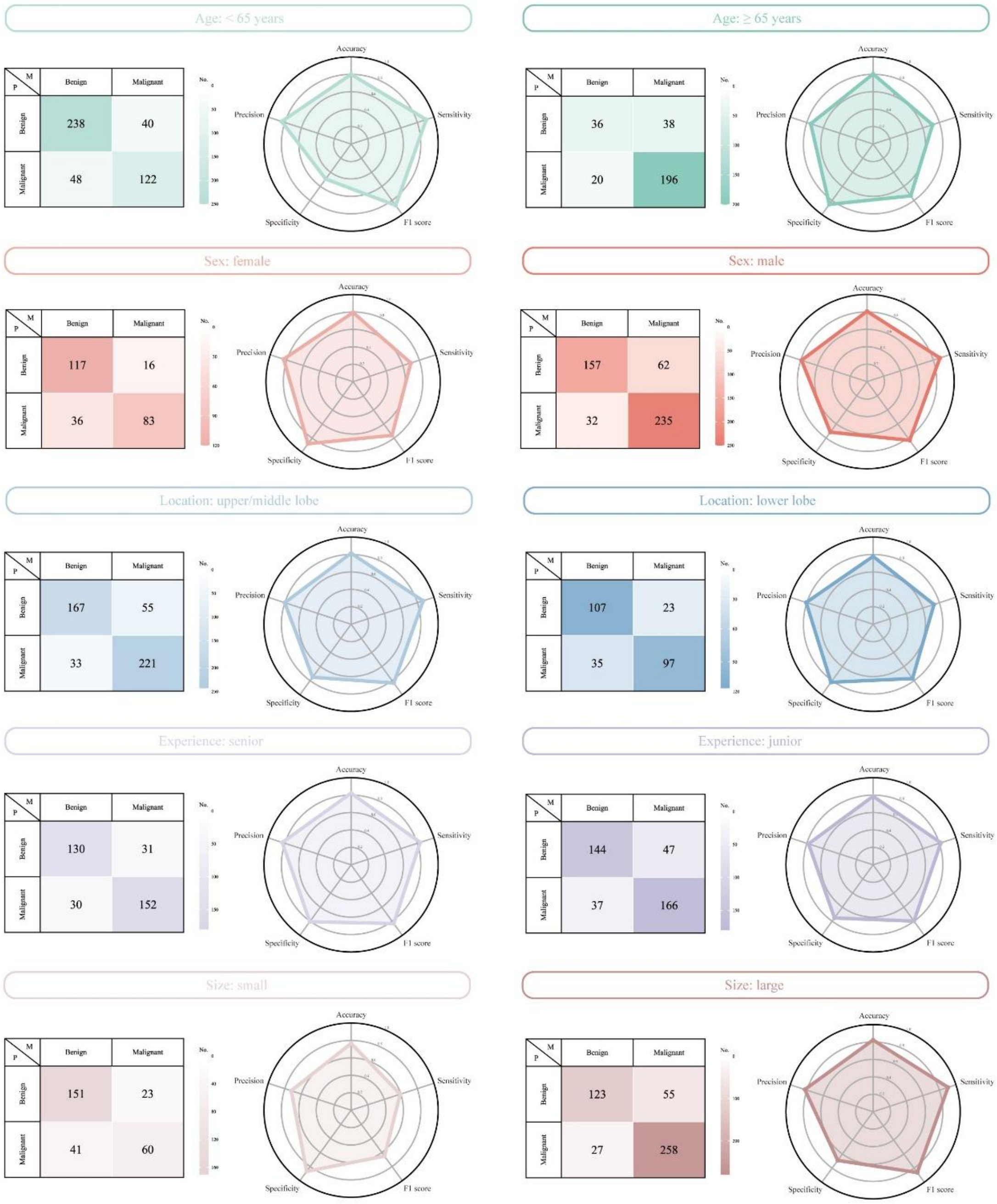

Supplement: Supplementary file 1 — ELECTRONIC SUPPLEMENTARY MATERIAL [file 13244_2026_2244_MOESM1_ESM.pdf]
